# Identification of potent human neutralizing antibodies against SARS-CoV-2 implications for development of therapeutics and prophylactics

**DOI:** 10.1038/s41467-021-25153-x

**Published:** 2021-08-09

**Authors:** Shaojuan Zhao, Huajun Zhang, Xinglou Yang, Haiwei Zhang, Ying Chen, Yancheng Zhan, Xiaoqing Zhang, Rendi Jiang, Meiqin Liu, Lan Liu, Li Chen, Wei Tang, Cheng Peng, Xiaoxiao Gao, Zhe Zhang, Zhengli Shi, Rui Gong

**Affiliations:** 1 CAS Key Laboratory of Special Pathogens and Biosafety, Wuhan Institute of Virology, Center for Biosafety Mega-Science, Chinese Academy of Sciences, Wuhan, Hubei China; 2 University of Chinese Academy of Sciences, Beijing, China

**Keywords:** Viral infection, SARS-CoV-2, Viral infection

## Abstract

Severe acute respiratory syndrome coronavirus 2 (SARS-CoV-2) is a novel coronavirus that is spreading rapidly, which seriously impacts global public health and economy. Thus, developing effective drugs remains urgent. We identify two potent antibodies, nCoVmab1 and nCoVmab2, targeting the SARS-CoV-2 spike protein receptor-binding domain (RBD) with high affinities from a naïve human phage-displayed Fab library. nCoVmab1 and nCoVmab2 neutralize authentic SARS-CoV-2 with picomolar and nanomolar IC_50_ values, respectively. No detectable defects of nCoVmab1 and nCoVmab2 are found during the preliminary druggability evaluation. nCoVmab1 could reduce viral titer and lung injury when administered prophylactically and therapeutically in human angiotensin-converting enzyme II (hACE2)-transgenic mice. Therefore, phage display platform could be efficiently used for rapid development of neutralizing monoclonal antibodies (nmabs) with clinical potential against emerging infectious diseases. In addition, we determinate epitopes in RBD of these antibodies to elucidate the neutralizing mechanism. We also convert nCoVmab1 and nCoVmab2 to their germline formats for further analysis, which reveals the contribution of somatic hypermutation (SHM) during nCoVmab1 and nCoVmab2 maturation. Our findings not only provide two highly potent nmabs against SARS-CoV-2 as prophylactic and therapeutic candidates, but also give some clues for development of anti-SARS-CoV-2 agents (e.g., drugs and vaccines) targeting the RBD.

## Abstract (web summary)

Applying neutralizing antibodies against SARS-CoV-2 to infected patients is a promising anti-viral treatment strategy. Here, Zhao et al. derive RBD-targeting monoclonal antibodies from a naïve human phage-displayed Fab library and show their  prophylactic and therapeutic potential in hACE2-transgenic mice.

## Introduction

Severe acute respiratory syndrome coronavirus 2 (SARS-CoV-2)^1^, which causes the disease known as COVID-19, is an emerging coronavirus^2,3^. It is associated with mild to severe respiratory disease, including lethal pneumonia. To date, SARS-CoV-2 continues to spread globally, with more than 180 million confirmed cases and a mortality rate of about 2% (https://www.who.int/emergencies/diseases/novel-coronavirus-2019/situation-reports). Although some drugs and vaccines have been approved for the treatment and prevention of SARS-CoV-2 infection, more antiviral agents are still urgently required. SARS-CoV-2 is a single-stranded positive-sense RNA virus that belongs to the genus Betacoronavirus (β-CoV) of the family Coronaviridae. It has a genome organization of a 5’ untranslated region (UTR), replicase complex (orf1ab), S gene, E gene, M gene, N gene, 3’ UTR, and 7 accessory genes^2^. As SARS-CoV, SARS-CoV-2 also utilizes angiotensin-converting enzyme II (ACE2) as its cellular receptor to enter the host^3^. The envelope protein of SARS-CoV-2, a typical class I viral fusion protein, is called spike protein (S protein) and contains two subunits,  S1 and S2, which are responsible for receptor binding and membrane fusion, respectively. The prefusion S protein is trimeric^4^ and contains a receptor-binding domain (RBD) on its S1 subunit^5^. Because the RBD is critical for viral entry and is highly antigenic^6^, it is an attractive target for drug and vaccine development. Numerous neutralizing monoclonal antibodies (nmabs) targeting the RBD have been developed for severe acute respiratory syndrome coronavirus (SARS-CoV)^7^ and Middle East respiratory syndrome coronavirus (MERS-CoV)^8^. These antibodies are potential therapeutics for clinical use^9^.

The crystal structure of the RBD in complex with hACE2 has been identified by different groups^5,10,11^. Compared with the SARS-CoV RBD, the hACE2-binding ridge in SARS-CoV-2 RBD binds to hACE2 more compactly, with the stabilization of two virus-binding hotspots at the RBD/hACE2 interface caused by several residue changes in the SARS-CoV-2 RBD^5^. These findings might indicate why SARS-CoV-2 has higher infectivity than SARS-CoV. Consequently, potent neutralizing antibodies that can block the RBD from binding to hACE2 require a high affinity.

Here, we report the identification of two neutralizing antibodies, nCoVmab1 and nCoVmab2, against SARS-CoV-2, from a large naïve human phage-displayed Fab library. Both of these antibodies could efficiently neutralize authentic SARS-CoV-2. nCoVmab1 also shows protective efficacy when administrated before and after the SARS-CoV-2 challenge in a hACE2-transgenic mouse model. In addition, we analyze the epitopes of nCoVmab1 and nCoVmab2 by alanine scanning to elucidate the neutralizing mechanism. We also investigate the binding abilities of the germline formats of nCoVmab1 and nCoVmab2 to the RBD, which preliminarily discloses the complexity and significance of the antibody maturation pathway for eliciting highly potent neutralizing antibodies by RBD.

## Results

### Identification of two candidate Fabs against the SARS-CoV-2 S protein RBD

**Fig. 1:**
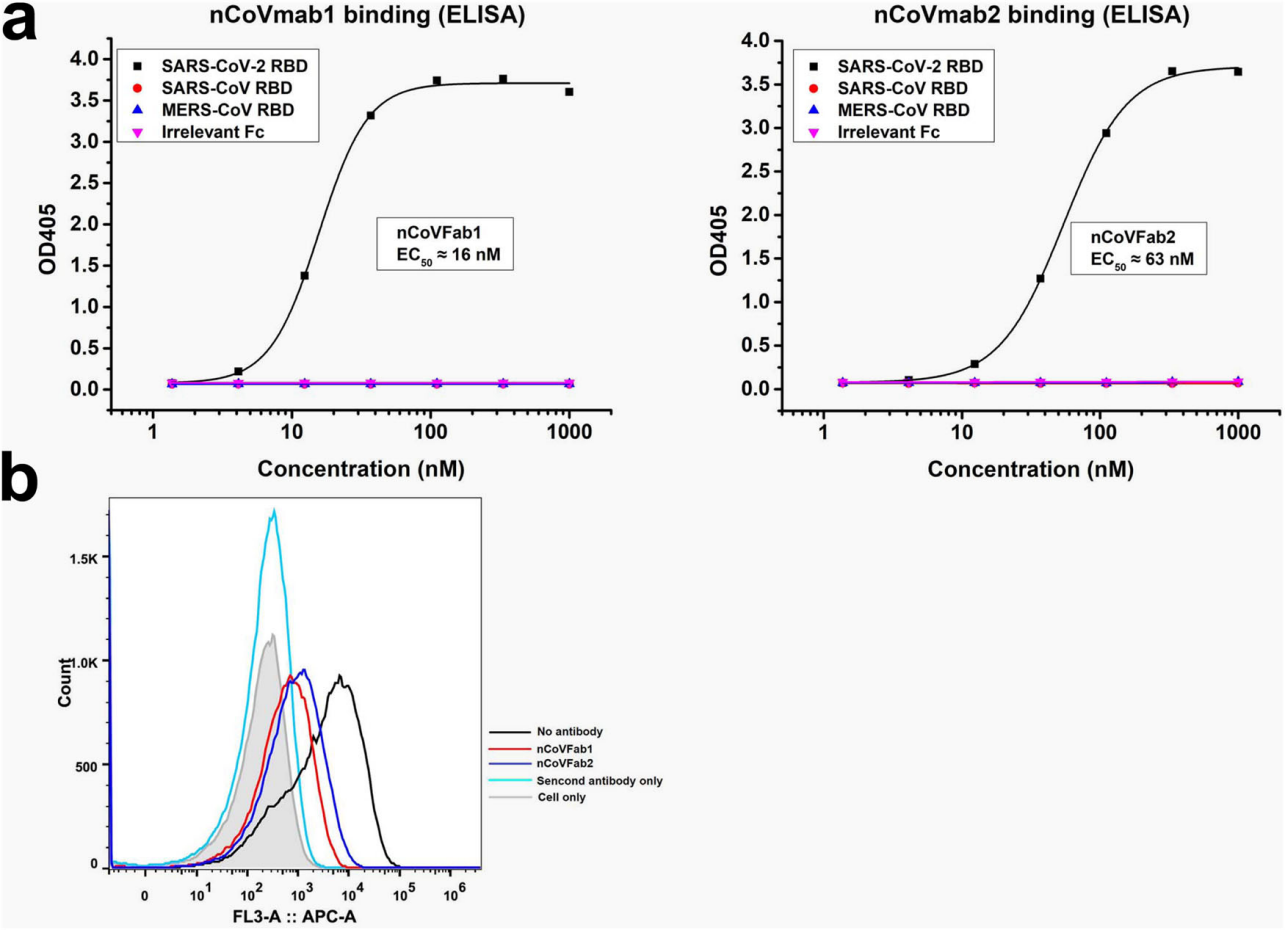
Binding of nCoVFab1 and nCoVFab2 to RBDs. **a** Binding of nCoVFab1 and nCoVFab2 to different RBDs, as measured by an ELISA. RBD-Fc fusions of SARS-CoV-2, SARS-CoV, and MERS-CoV were used as antigens. Irrelevant Fc protein was used as a negative control. The experiments were performed independently twice and similar results were obtained. One representative experiment is shown and data are average values of two replicates. **b** Blockage of binding of SARS-CoV-2 RBD-Fc to Vero E6 cells by Fabs. nCoVFab1 and nCoVFab2 inhibited the binding fluorescence shift with efficiencies of approximately 89% and 82%, respectively, as measured by flow cytometry.

In this study, we first constructed a SARS-CoV-2 RBD-Fc fusion protein for panning (Supplementary Fig. 1). An in-house constructed large naïve human phage-displayed Fab library was used for panning against biotinylated RBD-Fc with magnetic beads. After four rounds of panning, polyclonal phage ELISA was performed, followed by a monoclonal phage ELISA. Two enriched Fab clones (nCoVFab1 and nCoVFab2) were identified by sequencing. We first expressed these Fabs and tested their binding to RBD-Fc constructs from different coronavirus S proteins by ELISA (Fig. 1a). Both of them could specifically bind to the SARS-CoV-2 S protein RBD with EC_50_ values of ~16 nM for nCoVFab1 and ~63 nM for nCoVFab2, respectively. By contrast, neither could bind to the SARS-CoV and MERS-CoV S protein RBDs. In a functional assay, these two Fabs blocked the RBD from binding to the permissible Vero E6 cell line, as measured by flow cytometry (Fig. 1b and Supplementary Fig. 2). Therefore, these Fabs have the potential to inhibit SARS-CoV-2 infection.

### Characterization of nCoVmab1 and nCoVmab2

**Fig. 2:**
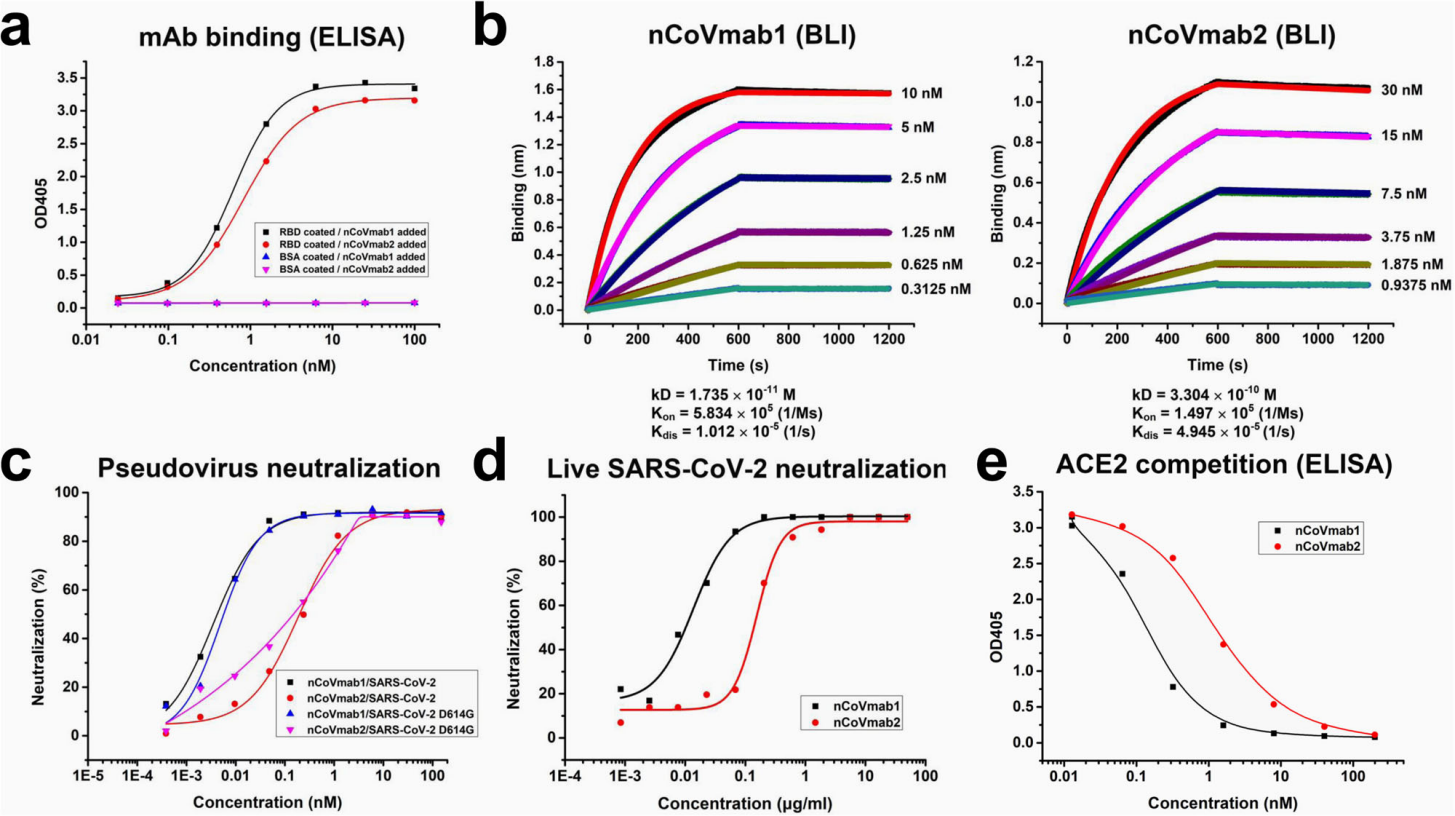
Activity identification of nCoVmab1 and nCoVmab2. **a** The binding of nCoVmab1 and nCoVmab2 to the RBD, as measured by an ELISA. Both nCoVmab1 and nCoVmab2 could specifically bind to the RBD antigen. The experiments were performed independently three times and similar results were obtained. One representative experiment is shown and data are average values of two replicates. **b** The kinetics of the binding of nCoVmab1 and nCoVmab2 to biotinylated RBD monitored by BLI. Fast association and slow dissociation were observed. **c** Neutralization of pseudotyped SARS-CoV-2 and SARS-CoV-2 D614G by nCoVmab1 and nCoVmab2. Inhibitory rates of infection were calculated by scanning fluorescent plaques. The experiments were performed independently twice and similar results were obtained. One representative experiment is shown and data are average values of two replicates. **d** Neutralization activity evaluation of nCoVmab1 and nCoVmab2 against live SARS-CoV-2. In a plaque assay, nCoVmab1 and nCoVmab2 efficiently blocked the interaction between the virus and host cell, with IC_50_ values of 0.010 and 0.139 μg/ml, respectively. The experiments were performed independently twice and similar results were obtained. One representative experiment is shown and data are average values of two replicates. **e** Competitive binding of the RBD to hACE2 by nCoVmab1 and nCoVmab2. The two antibodies could abolish 100% of the binding of the RBD to hACE2. The experiment was performed with duplicate samples. Data are average values of two replicates.

After nCoVFab1 and nCoVFab2 were converted to an IgG1 format (nCoVmab1 and nCoVmab2), ELISA was performed again. They could bind to coated RBD with EC_50_ values of 0.56 nM (nCoVmab1) and 0.84 nM (nCoVmab2) (Fig. 2a), respectively. The affinities of nCoVmab1 and nCoVmab2 to the RBD were confirmed by biolayer interferometry (BLI) technology. The affinities of nCoVmab1 and nCoVmab2 to the RBD were in the picomolar (1.735 × 10^−11^ M) and subnanomolar range (3.304 × 10^−10^ M), respectively in the case of labeling RBD with biotin (Fig. 2b). The measurement was also performed in a reverse manner with immobilization of antibodies on the sensor, which would allow for the accurate analysis of a monomeric binding interaction. The affinities were 8.375 × 10^−11^ M and 1.081 × 10^−9^ M in the case of nCoVmab1 and nCoVmab2, respectively (Supplementary Fig. 3). Hence, the avidity only has minor effects on the off-rate and affinity.

The neutralization activities of nCoVmab1 and nCoVmab2 were measured with the pseudotyped wildtype SARS-CoV-2. Notably, a SARS-CoV-2 variant carrying amino acid change D614G in S protein has become the most prevalent form in the early global pandemic^12^. D614G mutation has been reported to impact infectivity^13^ and favors an open conformational state of S protein^14^. Since it has been proved that D614G only enhances the viral infectivity but does not alter antigenicity of S protein and reduce the efficacy of vaccine^15^, it will be interesting to test whether it would reduce the antibody’s neutralizing activity. Therefore, pseudotyped SARS-CoV-2 with D614G mutation (SARS-CoV-2 D614G) has also been generated for neutralization tests to determine whether it could alter the epitope of the antibodies. nCoVmab1 and nCoVmab2 showed neutralization activities with half-maximal inhibitory concentration (IC_50_) values of 0.004 and 0.195 μg/ml against SARS-CoV-2, respectively, 0.006 and 0.165 μg/ml against SARS-CoV-2 D614G, respectively (Fig. 2c). In general, the neutralizing activities are not affected by D614G mutation. In a live viral plaque reduction assay, both nCoVmab1 and nCoVmab2 could neutralize SARS-CoV-2 live virus with high efficiency, with IC_50_ values of 0.010 μg/ml and 0.139 μg/ml, respectively (Fig. 2d); these results demonstrate extreme potency and are consistent with the affinity values. Additional antiviral experiments were performed to evaluate the potential for clinical use.

First, SARS-CoV-2 was incubated with the cells for 1 h, and then antibodies were added to inhibit virus plaque spread. No virus plaques were observed at nCoVmab1 and nCoVmab2 concentrations of 1.852 and 16.667 μg/ml, respectively (Supplementary Table 1). Second, after the antibodies were mixed with SARS-CoV-2, the mixture was added to cells for cytopathic effect (CPE) observation after 48 h without removing the supernatant. nCoVmab1 and nCoVmab2 could completely neutralize the virus, without an observed CPE, at 0.617 and 5.556 μg/ml, respectively (Supplementary Table 2). We also found that both nCoVmab1 and nCoVmab2 could competitively block the binding of the RBD to hACE2 (Fig. 2e), which confirmed the neutralizing mechanism.

To further evaluate efficiency in an animal model, we firstly examined the clinical potential of these two antibodies. Since the autoreactivity of an antibody is an important consideration in terms of application in vivo, we performed a binding assay with HEp-2 cells, a human epithelial cell line^16^, and found that our antibodies did not display observable binding to HEp-2, which was in contrast to 2F5, a broadly reactive anti-HIV-1 envelope gp41 human mAb that is a polyspecific autoantibody reacting with the phospholipid cardiolipin^17,18^ (Supplementary Fig. 4). The cytotoxicity of these two antibodies was also tested in five types of cells from humans, monkeys, and mice, and no detectable toxicity was observed (Supplementary Fig. 5). In addition, we found that both nCoVmab1 and nCoVmab2 were stable when incubated with mouse sera at 37 °C after 20 days (Supplementary Fig. 6). Therefore, at least one monoclonal antibody, nCoVmab1, could be tested in an animal model to verify whether it could be a promising therapeutic candidate for clinical use.

### Protective efficacy of nCoVmab1 in a hACE2-transgenic mouse model

**Fig. 3:**
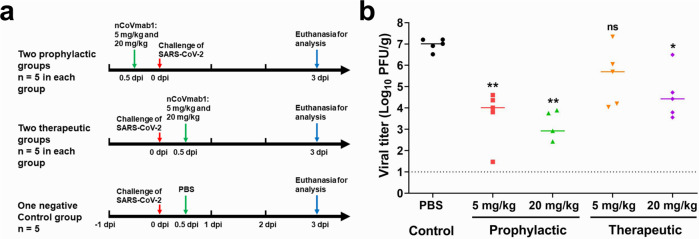
Protective efficacy of nCoVmab1 in vivo. **a** Experimental route of prophylactic and therapeutic tests of nCoVmab1 in hACE2 transgenic mice. hACE2 transgenic mice were intraperitoneally injected with low (5 mg/kg, *n* = 5 mice/group) and high (20 mg/kg, *n* = 5 mice/group) doses of nCoVmab1 12 h before or 12 h after SARS-CoV-2 infection. PBS was used as a negative control (*n* = 5 mice/group). **b** The viral titers in the lungs from different groups were determined at 3 dpi by plaque assay (dashed line represents the detection limit). The median values were presented. In the high-dose prophylactic group, one mouse had a viral titer below the detection limit. Statistical significance was measured by using one-way ANOVA with Dunnett’s multiple comparisons, not significant [ns]: *p* > 0.05, *p < 0.05, **p < 0.01. For the prophylactic experiment in **b**, *p* value between the PBS group and the low dose group is 0.0096; *p* value between the PBS group and the high dose group is 0.0095. For the therapeutic experiment in **b**, *p* value between the PBS group and the low dose group is 0.2007; *p* value between the PBS group and the high dose group is 0.0150.

nCoVmab1 was tested in vivo for its protective efficacy using the hACE2-transgenic mouse model described previously^19^. The mice were intraperitoneally injected with a single dose of 5 or 20 mg/kg of nCoVmab1 12 h before or 12 h after an intranasal challenge of 1 × 10^4^ TCID_50_ SARS-CoV-2 (Fig. 3a).

Viral titers in the lung were monitored 3 days post-infection (dpi). In the prophylactic groups with the administration of low-dose and high-dose antibodies, the viral titers decreased about 1000 and 10000 times, respectively, while in the treatment groups, the viral titers decreased about 10 (low dose) and 100 (high dose) times (Fig. 3b).

**Fig. 4:**
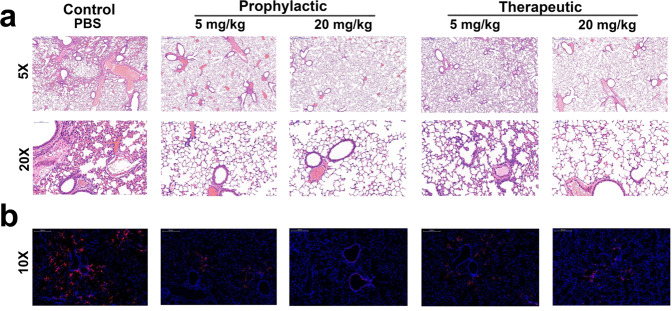
Pathological changes of lung sections. **a** Pathological changes of H&E-stained lung sections from the prophylactic and therapeutic groups (*n* = 5 mice/group). No obvious lung lesions were observed in the high-dose prophylactic groups compared with the PBS group. **b** Viral antigens in mice of the prophylactic and therapeutic group were tested by IFA (*n* = 5 mice/group). Viral antigens (red dot) were detected by anti-2019-nCoV NP protein polyclonal antibody. Almost no obvious viral antigens were detected in the high-dose prophylactic groups compared with the PBS group, which was in accordance with the results from the H&E-staining. The images and areas of the H&E-stained sections were magnified by ×5 (scale bars, 500 μm) and ×20 (scale bars, 100 μm). The images and areas of sections from IFA were magnified by ×10 (scale bars, 200 μm).

Histological analysis was also performed on the lungs from mice that were administered nCoVmab1 12 h pre-infection and 12 h post-infection at 3 dpi (Fig. 4a). After hematoxylin-eosin (H&E) staining, the lungs from the PBS group displayed lung pathology with increased inflammatory cells around blood vessels and branches, extensive alveolar wall broadening and thickening, prominent inflammatory cells infiltration, and a small amount of exudation. For the low-dose prophylaxis group that received 5 mg/kg of nCoVmab1, the lung pathology was characterized by a slight increase in perivascular inflammatory cells. The lung pathology displayed no essential lesions in the high-dose prophylactic group that received 20 mg/kg of nCoVmab1. For the low-dose therapeutic group that received 5 mg/kg of nCoVmab1, the lung pathology showed a slight increase in perivascular inflammatory cells, alveolar wall widened and slightly thickened. The lung pathology showed only a slight increase in perivascular inflammatory cells in the high-dose therapeutic group that received 20 mg/kg of nCoVmab1. These data demonstrate that nCoVmab1 could reduce lung pathology following SARS-CoV-2 infection, which is in accordance with the change in viral titers.

To further confirm the effect of protection caused by the reduction of viral replication in the lungs, viral antigens were detected by indirect immunofluorescence assays (IFA) as previously described^3,20^. There were only a few viral antigens stained in the high-dose prophylaxis group compared with that in the PBS group, and viral antigens in other groups also significantly decreased (Fig. 4b).

Taken together, these data indicate that nCoVmab1 can efficiently prevent SARS-CoV-2 infection in vivo and can significantly inhibit viral replication in mice after viral inoculation. These results demonstrate the in vivo efficacy of nCoVmab1, indicating its potential prophylactic and therapeutic applications in humans against SARS-CoV-2 infection.

### Epitope determination

**Fig. 5:**
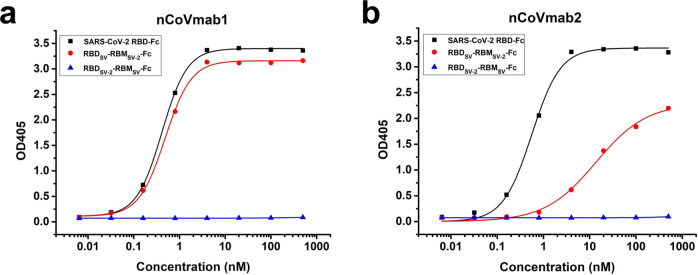
Determination of the epitopes of nCoVmab1 and nCoVmab2. Binding of nCoVmab1 (**a**) and nCoVmab2 (**b**) to chimeric RBD-Fc fusions. nCoVmab1 could bind to the SARS-CoV-2 RBD and RBD_SV_-RBM_SV-2_ with similar binding activities whereas the binding ability of nCoVmab2 to RBD_SV_-RBM_SV-2_ was much lower than that of nCoVmab1. Therefore, both antibodies primarily recognized the RBM in the SARS-CoV-2 RBD. The experiment was performed with duplicate samples. Data are average values of two replicates.

To determine the epitope regions of these two antibodies, we constructed chimeric RBDs for testing (Supplementary Fig. 1). The receptor-binding motif (RBM) in the SARS-CoV-2 RBD was replaced by the SARS-CoV RBM (RBD_SV-2_-RBM_SV_), whereas the SARS-CoV RBM was substituted with the SARS-CoV-2 RBM (RBD_SV_-RBM_SV-2_). We found that both nCoVmab1 and nCoVmab2 could only bind to the SARS-CoV-2 RBD and RBD_SV_-RBM_SV-2_, which indicates that the major residues involved in the antibody/antigen interaction are located in the RBM in the SARS-CoV-2 RBD (Fig. 5). Notably, the binding of nCoVmab1 to the RBD_SV_-RBM_SV-2_ was similar to that to the SARS-CoV-2 RBD, whereas the binding of nCoVmab2 to the RBD_SV_-RBM_SV-2_ decreased compared with that to the SARS-CoV-2 RBD. The change of binding indicates that the epitopes of these two antibodies might be slightly different although the competition ELISA with these two antibodies showed that their epitopes overlapped (Supplementary Fig. 7).

To further refine the epitopes of nCoVmab1 and nCoVmab2, a panel of RBD mutants with single mutations involved in ACE2 binding^5,10^ were generated by alanine-scanning in the RBM region. The binding of nCoVmab1, nCoVmab2, and ACE2 to the RBD mutants was measured by ELISA (Supplementary Fig. 8a). The binding of ACE2 to most RBD mutants significantly declined, while much less mutants showed decreased binding to nCoVmab1 and nCoVmab2 (Table 1 and Supplementary Fig. 8b). The identified mutations in RBD that could reduce the binding to these two mabs also decreased the binding to ACE2, while quite a few mutations in RBD that could abort the binding to ACE2 did not alter the binding to the antibodies. In summary, the epitope analysis confirms that nCoVmab1 and nCoVmab2 inhibit the viral infection by competitive binding to RBD with hACE2, like other reported nmabs such as CB6^21^, B38^22^, and CV30^23^. The epitopes of these antibodies share some key residues in RBD (e.g., Y453/L455/F456/N487/Y489).

**Table 1:** Fold change in EC_50_ binding activity compared to wild type.

Residue mutation	Fold change in EC_50_ compared to wild-type		
	ACE2	nCoVmab1	nCoVmab2
WT	1^a^	1	1
G446	>13.3	1.08	1.76
Y449	–^b^	0.93	1.27
Y453	–	–	–
L455	>14.6	>71.97	–
F456	–	–	–
F486	–	0.97	1.17
N487	–	>1.74	>9.21
Y489	–	–	–
Q493	0.98	1.04	1.37
G496	>15.8	1.04	1.15
Q498	>3.6	1.06	1.07
T500	–	1.06	1.56
N501	>5.29	1.13	>3.72
G502	–	>8.04	0.96
Y505	–	1.15	>3.29

^a^Number in the column represents the fold change in EC_50_ of binding to RBD mutant relative to the wildtype RBD (WT). The value (>1.5) is defined as the impact on binding.

^b^“–” indicates no binding. The OD405 value less than 0.5 at the highest concentration of antibody is defined as no binding.

### Germline format analysis

nCoVmab1 and nCoVmab2 share the same complementarity-determining regions (CDRs) in the heavy chain variable domain (VH), while only several residues differ in the framework regions. However, they have different light chain variable domains (VLs). Notably, the binding and neutralization activities of nCoVmab1 are approximately 10 times higher than those of nCoVmab2. Therefore, the light chain is also important for achieving high efficacy. We further analyzed the germline information of both nCoVmab1 and nCoVmab2 (Table 2) by IMGT/V-QUEST^24,25^. In general, both nCoVmab1 and nCoVmab2 are germline-like antibodies although they have somatic hypermutations (SHMs) compared with their germline format, which is desired because of our library construction strategy. With more details, their VHs exhibit a low SHM rate compared with that of their germline genes (nucleotide identity (V_H _+ J_H_) = 97.98% for nCoVmab1, nucleotide identity (V_H _+ J_H_) = 97.40% for nCoVmab2). By contrast, the VL of nCoVmab1 undergoes relatively more SHMs, with an identity of 91.61% (V_L _+ J_L_), while the VL of nCoVmab2 has fewer SHMs, with an identity of 98.45% (V_L _+ J_L_). It has been reported that quite a lot of the neutralizing antibodies against SARS-CoV-2 belong to IGHV3-53 or IGHV3-66^21–23,26–30^. We found that nCoVmab1 and nCoVmab2 were encoded by the IGHV3-66 as well. The amino acid sequences of V_H_ genes of these antibodies were similar (Supplementary Fig. 9), and there were only several amino acid differences in the heavy chains CDR1 (HCDR1s) and CDR2 (HCDR2s), whereas their heavy chain CDR3s (HCDR3s) were quite different (Supplementary Table 3). Therefore, the nmabs targeting RBD in SARS-CoV-2 S protein may prefer to use IGHV3-53 and IGHV3-66 although they were derived from different resources. In addition, these antibodies have different light chains (Supplementary Table 3). These diversities could further refine the binding sites and determine the activities of the nmabs.

**Table 2:** Germline analysis of nCoVmab1 and nCoVmab2.

mAbs	V_H_	D_H_	J_H_	Identity (V_H_)	Identity (J_H_)	V_L_	J_L_	Identity (V_L_)	Identity (J_L_)
nCoVmab1	IGHV3 -66*02	IGHD3 -10*01	IGHJ 6*02	98.95% (282/285 nt)	93.55% (58/62 nt)	IGLV1 -40*02	IGLJ 3*02	90.97% (262/288 nt)	97.06% (33/34 nt)
nCoV mab2	IGHV3 -66*02	IGHD3 -10*01	IGHJ 6*02	98.25% (280/285 nt)	93.55% (58/62 nt)	IGLV1 -40*01	IGLJ 3*02	98.26% (283/288 nt)	100.00% (33/34 nt)

V-(D)-J rearrangement summary for nCoVmab1 and nCoVmab2. Listed data are the top matches for *Homo sapiens* germline genes corresponding to the V, D, and J of the heavy chain (V_H_, D_H_, and J_H_) and the V and J of the light chain (V_L_ and J_L_). Nucleotide sequence identities with the top-matched VH and VL genes are also listed.

**Fig. 6:**
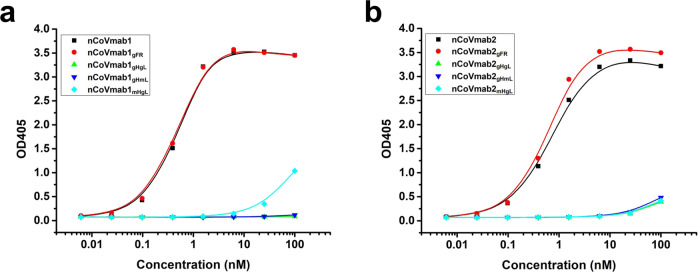
Recognition of the RBD by the germline formats of nCoVmab1 and nCoVmab2. **a** nCoVmab1 and its germline formats. **b** nCoVmab2 and its germline formats. The SHMs on the FR did not change the binding activities in either nCoVmab1 or nCoVmab2. The SHMs on the CDRs dramatically reduced binding. For nCoVmab1, maturation of the heavy chain was important for the acquisition of preliminary binding activities. In nCoVmab2, the germline format might initiate binding. The synergetic maturation of both the heavy and light chains was crucial for high-affinity binding. The experiment was performed with duplicate samples. Data are average values of two replicates.

To determine the role of SHM during affinity maturation, we converted nCoVmab1 to its germline format with different heavy and light chain combinations (Supplementary Fig. 10). We constructed nCoVmab1_gHgL_ (germline heavy chain and germline light chain), nCoVmab1_mHgL_ (mature heavy chain and germline light chain), and nCoVmab1_gHmL_ (germline heavy chain and mature light chain). Moreover, we also prepared a nCoVmab1 mutant (nCoVmab1_gFR_) by converting the framework regions (FRs) to the corresponding germline sequence and reservation of the CDRs as their mature status. The binding of nCoVmab1_gFR_ to RBD did not change compared to that of nCoVmab1, which indicates that mutations in FRs will not affect the recognition of antibodies/antigens in this case. However, no obvious binding was observed for either nCoVmab1_gHgL_ or nCoVmab1_gHmL_, whereas, modest binding was observed for nCoVmab1_mHgL_ (Fig. 6a). We also constructed serial germline formats for nCoVmab2 as we had for nCoVmab1. In this case, nCoVmab2_gFR_ displayed binding activity as strong as that of nCoVmab2. nCoVmab2_gHgL_, nCoVmab2_mHgL_, and nCoVmab2_gHmL_ weakly bound to the RBD (Fig. 6b). Therefore, in our case, the SHMs in FRs have no obvious effect on binding, while the SHMs in CDRs are very important for the achievement of high binding.

## Discussion

The nmabs are powerful weapons to fight against infectious diseases. To date, a panel of neutralizing antibodies from transgenic mice and convalescent patients from SARS-CoV-2 infections have been identified and characterized^31,32^. Among these antibodies, some of them have already received FDA emergency use authorization for the treatment of COVID-19^33,34^. Here, we report two potent human neutralizing antibodies, nCoVmab1 and nCoVmab2, against SARS-CoV-2. These antibodies were selected from a large naïve human phage-displayed Fab library. They show high neutralizing potency against pseudotyped SARS-CoV-2, while they also show similar neutralizing activities against pseudotyped SARS-CoV-2 D614G. Notably, one antibody, nCoVmab1, shows exceptional neutralizing activity against authentic SARS-CoV-2 by blocking the receptor binding to host cells. The other antibody, nCoVmab2, is also good at neutralizing the virus. In addition, these two antibodies can inhibit virus plaque spread and CPE occurrence, and no detectable drawbacks were found during the stability, autoreactivity, and cytotoxicity tests of these two antibodies. In addition, nCoVmab1 was further evaluated in a K18-hACE2 transgenic mouse model. nCoVmab1 shows good prophylactic and therapeutic effects with reduction of lung viral titers by 10-10000-fold, as well as significant protection of lung lesions. Overall, by using a phage display platform, we can produce candidate nmabs with clinical potential against emerging infectious diseases without special animal sources (e.g., transgenic mice) or samples (e.g., blood samples from convalescent patients).

The RBD is the primary choice as a target for the development of neutralizing antibody candidates against coronaviruses. In a previous study, mAbs have been identified by using RBD from SARS-CoV and MERS-CoV as antigens^35,36^. The antibody can neutralize the virus by directly occupying the binding sites of the receptor on the RBD^37^ or inducing a conformational change in the RBD to abort RBD binding to the receptor^38^. Our neutralizing antibodies nCoVmab1 and nCoVmab2 competitively interact with SARS-CoV-2 S protein RBD and subsequently abort the binding between RBD and hACE2. According to the alanine scanning, the amino acids in RBD involved in binding have been revealed. The information is helpful for understanding the mechanism of escaping from immune surveillance by neutralizing antibodies and selecting certain residues as targets for the development of broad antiviral agents against SARS-CoV-2 variants.

nCoVmab1 and nCoVmab2 are germline-like antibodies with total identities (V_H _+ J_H _+ V_L _+ J_L_) of 94.92% and 97.76%, respectively. It might also demonstrate that RBD could induce high potency antibodies in vivo without a complex antibody maturation pathway. Therefore, the RBD could be a subunit vaccine candidate against SARS-CoV-2 due to its high immunogenicity and the RBD-based subunit vaccine has received emergency use authorization^39–43^. In addition, the germline-like antibody has less mutations compared with the corresponding matured antibody, which may bring benefits when the administration in vivo and causes attention now^44,45^.

Although nCoVmab1 and nCoVmab2 share very similar heavy chains, the completely different light chains might lead to differences in affinity and neutralizing activity. The SHM rate of the heavy chain is very low. By contrast, nCoVmab1, which is much more potent than nCoVmab2, has more SHMs in its light chain. The V_H_ genes of nCoVmab1 and nCoVmab2 are encoded by IGHV3-66 genes. Interestingly, the V_H_ genes of a panel of currently identified nmabs with high potency also belong to IGHV3-53 or IGHV3-66. It seems the nmabs targeting RBD preferentially use V_H_ genes from IGHV3-53 or IGHV3-66. The mechanism needs to be further investigated. The RBD recognition by the nCoVmab1, nCoVmab2, and their germline formats disclose not only the possibilities but also the complexities of the elicitation of efficient neutralizing immune responses targeting the RBD. Our results could be helpful for further designing vaccines based on the RBD.

SARS-CoV-2 is still quickly spreading worldwide. Continuous work on the selection of more antiviral candidates is still extremely important. Effective drugs that could provide more clinical benefits for the patients are highly desired. Our antibodies reported here are also translatable as potential therapeutics and prophylactics for fighting SARS-CoV-2 infection.

## Methods

### Ethics statements

Twenty-five male mice (eight to ten weeks old) were randomly assigned to different groups in this study. Viral infections were performed according to the standard operating procedures of the biosafety level 3 (BSL-3) facility. All processes of the animal experiment were in line with recommendations for the care and use of laboratory animals, and the Institutional Review Board of the Wuhan Institute of Virology, Chinese Academy of Sciences (Ethics number: WIVA21202002).

### Cells and virus

Vero (catalog no. CCL-81, ATCC), Vero E6 (catalog no. GDC146, CCTCC), 293T cells (catalog no. GDC187, CCTCC), L-929 (catalog no. GDC034, CCTCC), HEp-2 (catalog no. GDC004, CCTCC), and Huh7 cells (catalog no. GDC134, CCTCC) were cultured in DMEM (Gibco) with 10% FBS (Gibco), 100 units/ml penicillin, and 100 μg/ml streptomycin (Gibco) at 37 °C and 5% CO_2_. OVCAR-3 cells (catalog no. TCHu228, CCTCC) were cultured in RPMI Medium 1640 (Gibco) with 20% FBS at 37 °C and 5% CO_2_. HEK293F cells (Thermo) were cultured using Freestyle 293 expression medium (Invitrogen) in an incubator at 37 °C and 8% CO_2_ with shaking at 180 rpm/min. Severe acute respiratory syndrome coronavirus 2 (SARS-CoV-2, strain: IVCAS 6.7512) was offered by the National Virus Resource, Wuhan Institute of Virology, Chinese Academy of Science. All processes in this study involving authentic SARS-CoV-2 were performed in a biosafety level 3 (BSL-3) facility.

### hACE2 transgenic mice

Heterozygous transgenic mice expressing the human ACE2 receptor driven by the epithelial cell cytokeratin-18 gene promoter (K18-hACE2)^19^. The K18-hACE2 model of SARS-CoV-2 infection recapitulates many features of severe COVID-19 infection in humans and can be used to define the mechanistic basis of lung disease and test immune and antiviral-based countermeasures.

### Preparation of the RBD and hACE2 recombinant proteins

The genes encoding the SARS-CoV-2 S protein RBD (GenBank: QHR63250.2, residues 319–541), SARS-CoV S protein RBD (GenBank: AAP30030.1, residues 306–527), and MERS-CoV S protein RBD (GenBank: AFS88936.1, residues 377–588) were constructed in the expression vector pCAGGS. The chimeric RBDs were also constructed as recently reported^5^. The constructs contained a signal peptide at the N-terminus and an IgG1 Fc fragment at the C-terminus. In addition, a thrombin site was inserted between the RBD and Fc fragments. If there is no special indication, RBD indicates the SARS-CoV-2 RBD. Using the same strategy, the encoding recombinant hACE2 (GenBank: NP068576.1, residues 19–615), hACE2-Fc, was also constructed for expression. All proteins were prepared as previously described^46^. Briefly, the plasmids were transfected into FreeStyle 293 cells (293F). After five days, the supernatant was collected and purified with Protein A resin (GE Healthcare). The purified proteins were concentrated with a 10-kDa centrifugal filter device (Millipore), and the concentrations were measured by a NanoPhotometer N60 (Implen) with the corresponding extinction coefficient. In addition, part of the purified RBD-Fc protein of SARS-CoV-2 was further digested by thrombin (Sigma-Aldrich) to obtain isolated RBD without the Fc fragment.

### Selection of specific Fab clones targeting the SARS-CoV-2 RBD

According to the EZ-link^®^Sulfo-NHS-LC-Biotin (Pierce) reagent instructions, the RBD-Fc was incubated with 10 mM biotin reagent at 37 °C for 30 min and then the reaction buffer was replaced with PBS. The labeled protein was tested by ELISA. A large naive human phage-displayed Fab library constructed in-house was panned against biotinylated RBD-Fc with Dynabeads™ Streptavidin Beads (Invitrogen)^47^. After multiple rounds of screening, a polyclonal phage enzyme-linked immunosorbent assay (ELISA) was performed, followed by a monoclonal phage ELISA to identify positive clones. For the phage ELISA, purified RBD-Fc at a concentration of 4 μg/ml was coated on the plate (Corning) at 4 °C overnight, and an irrelevant protein containing the Fc fragment was also coated as a control. The polyclonal phage from each round and the monoclonal phage were prepared, added to the wells, and incubated at 37 °C for 90 min according to standard protocols. An HRP-conjugated anti-M13 mouse monoclonal antibody (HRP) (Sino Biological) was used as the secondary antibody. ABTS (Invitrogen) was used as the substrate for color development. The selected Fab clones were sequenced, and the two enriched clones were named nCoVFab1 and nCoVFab2. They were expressed in *E. coli* strain HB2151, purified with nickel-nitrilotriacetic acid resin (Qiagen), and concentrated by a 3-kDa centrifugal filter device (Millipore).

### Binding of the two Fabs to different RBDs

The binding of these two Fabs to different RBDs from SARS-CoV-2, SARS-CoV, and MERS-CoV was evaluated by an ELISA. RBD-Fc fusions from SARS-CoV-2, SARS-CoV, and MERS-CoV were coated as described above. Irrelevant Fc protein also coated a negative control. Threefold serially diluted Fabs from 1000 nM were added and incubated at 37 °C for 90 min. A mouse monoclonal ANTI-FLAG^®^ M2-Peroxidase (HRP) antibody (Sigma-Aldrich) was used as the secondary antibody.

### Blockage of RBD binding to sensitive cells by the two Fabs

Flow cytometry was used to test whether the two Fabs could inhibit the binding of the RBD to the sensitive cell line. Briefly, Vero E6 cells were disrupted with sodium citrate (dissolved in PBS) and aliquoted into tubes. The Fabs (final concentration: 100 μg/ml) were incubated with RBD-Fc (final concentration: 2.5 μg/ml) at 4 °C for 1 h, and then the mixture was added to 60 μl of cells for incubation at 4 °C for 1.5 h. The cells were washed once with PBS. Goat anti-human IgG Fc-DyLight 650 (Invitrogen) was used as a secondary antibody for incubation at 4 °C for 1 h. The cells were washed again with PBS. Finally, the cells were resuspended in 200 μl of PBS for analysis on a CytoFLEX (Beckman Coulter).

### Preparation of nCoVmab1 and nCoVmab2

The Fab clones nCoVFab1 and nCoVFab2 were converted to IgG1 formats termed nCoVmab1 and nCoVmab2 for further characterization. The intact DNA sequences of nCoVmab1 and nCoVmab2 were cloned into the vector pVITRO2-neo-mcs (InvivoGen). IgG1s were expressed in 293F cells and purified with Protein A resin (GE Healthcare). The purified proteins were concentrated with a 10-kDa centrifugal filter device (Millipore).

### Binding of nCoVmab1 and nCoVmab2 to the RBD

These two IgGs were first evaluated for their RBD-binding abilities. Fourfold serial diluted IgGs from 100 nM were added and incubated at 37 °C for 90 min. Anti-human IgG (Fab specific)-peroxidase (Sigma-Aldrich) was used as the detection antibody.

### The affinity of nCoVmab1 and nCoVmab2 for the RBD

The affinity between the antigen and antibodies was measured by biolayer interferometry (BLI) using the Octet RED96 system (ForteBio). This experiment was performed at room temperature. According to the EZ-link^®^Sulfo-NHS-LC-Biotin (Pierce) reagent instructions, the RBD was biotinylated. All protein samples were diluted in PBS containing 0.01% Tween-20 and 0.1% BSA. The biosensors were pre-balanced in the buffer for 300 s. Biotinylated RBD at a concentration of 40 μg/ml was loaded on the biosensors for 200 s and flowed with twofold diluted IgG1s (nCoVmab1 was diluted from 10 nM to 0.31 nM and nCoVmab2 was diluted from 30 nM to 0.93 nM) for 600 s, and the probe was soaked in the buffer for 600 s to measure dissociation. After each reaction, the binding and dissociating states of the antibodies were easily evaluated, and the dissociation equilibrium constant was calculated using Data Analysis 7.0 software. The assay was also performed in a reverse manner. The biotinylated mAbs at a concentration of 40 μg/ml, were loaded on the biosensors for 200 s and flowed with twofold diluted RBD for 600 s, and the probe was soaked in the buffer for 600 s to measure dissociation.

### Inhibition of pseudotyped SARS-CoV-2 Infection

At first, the neutralizing activities of antibodies were performed by the Vesicular stomatitis virus (VSV) pseudovirus system. These pseudoviruses were restricted to a single round of replication, thus enabling them to be performed in a BSL-2 lab. Briefly, wildtype SARS-CoV-2 S gene and the D614G variant were constructed in vector pCAGGS. 293T cells were seeded at a density of 5 × 10^5^ cells/well in a 6-well plate overnight. The plasmids encoding the SARS-CoV-2 S gene and the S gene with D614G mutation were transfected into 293T cells by Lipofectamine™ 3000 according to the instruction (Invitrogen). The plate was cultured at 37 °C for 24 h, and then the transfected 293T cells were infected with VSVΔG-GFP/VSV G at MOI = 3 for 2 h^48^. The supernatant was replaced by DMEM with 2% FBS for culturing at 37 °C for 24 h. The supernatant containing pseudovirus (VSVΔG-GFP/SARS-CoV-2 S) was collected and clarified by centrifugation at 1500×*g* for 5 min. The virus titer was tested by infecting Vero E6 cells. Five-fold serially diluted nCoVmab1 and nCoVmab2 were mixed with pseudoviruses for incubation at 37 °C for 1 h. The mixture was added to the 96-well plates containing Vero E6 cells for incubation at 37 °C for 24 h. The plates were imaged in Operetta CLS (PerkinElmer) to obtain fluorescent plaques. The inhibitory effects of antibodies against pseudotyped SARS-CoV-2 were analyzed by Harmony High-Content Imaging and Analysis Software (PerkinElmer).

### Neutralization tests of nCoVmab1 and nCoVmab2

A live SARS-CoV-2 neutralization assay was first performed to evaluate the blockage of virus attachment by antibodies with a plaque assay in a biosafety level 3 laboratory. In brief, Vero cells were seeded at 1.5 × 10^5^ per well in 24-well culture plates overnight. Both nCoVmab1 and nCoVmab2 samples were threefold serially diluted in DMEM with 2.5% FBS. An equal volume including 300 PFU/ml SARS-CoV-2 was added, and the antibody-virus mixture was incubated at 37 °C for 1 h. Then, half of the mixture was added to a 24-well culture plate containing Vero cells. The cells infected with 150 PFU/ml SARS-CoV-2 only and those without the virus were used as the positive and uninfected controls, respectively. After the sample plates were incubated at 37 °C for 1 h and the antibody-virus mixture was removed, the Vero cell surface was overlaid with 1 ml of DMEM with 2.5% FBS plus 0.9% carboxymethyl cellulose for further incubation at 37 °C with 5% CO_2_ for 3 days. Plaques were clearly observed after 0.5% crystal violet staining. The curve was fitted to a sigmoid to calculate the neutralizing effect (IC_50_).

The microantiviral spread assay (I) is an effective method for observing the cytopathic effect (CPE) to detect antibody neutralizing activity^49^. Vero E6 cells were seeded in 96-well plates at a density of 1 × 10^4^ cells per well overnight. The supernatant was removed, and 200 TCID_50_ of SARS-CoV-2 was added. After incubation at 37 °C for 1 h, the virus supernatant was removed and washed with PBS. Threefold serially diluted nCoVmab1 and nCoVmab2 were incubated for 48 h at 37 °C. Cells infected with 200 TCID_50_ of SARS-CoV-2 and cells without the virus were used as the positive and uninfected controls, respectively. Simultaneously, remdesivir (gift from MedChemExpress LLC), which can inhibit SARS-CoV-2 replication^50^, was also used as a control. Then, the inhibition of viral replication was determined by observing the CPE with optical microscopy. In another microneutralization experiment (II), the antibody/virus mixture was continuously incubated with Vero E6 cells. Threefold serially diluted nCoVmab1 and nCoVmab2 were mixed with 150 TCID_50_ of SARS-CoV-2 and incubated at 37 °C for 1 h. Then, the antibody/virus mixture was transferred to Vero E6 cells. After incubation at 37 °C for 48 h, the CPE was observed.

### Competitive ELISA for determining the neutralization mechanism

A competitive ELISA was used to test the competitive binding of nCoVmab1 and nCoVmab2 to the RBD, which could reveal the neutralization mechanism of the two antibodies. Briefly, hACE2-Fc was coated on a plate. nCoVmab1 and nCoVmab2 were fivefold serially diluted and mixed with biotinylated RBD at a fixed concentration of 0.25 nM. Then, the mixture was added to the hACE2-coated wells. HRP-conjugated streptavidin (Proteintech) was used as the secondary antibody. The following procedures were performed as described above.

### Stability test

Purified nCoVmab1 and nCoVmab2 were mixed with equal volumes of mouse serum (Lablead) at a final antibody concentration of 1 mg/ml. The samples at different time points were removed, diluted in PBS, and frozen at −20 °C for further analysis. Samples at different time points (0, 1, 4, 8, 12, 16, and 20 days) were tested at a final antibody concentration of 0.33 nM, as calculated from the initial mixture by an ELISA. In addition, samples from day 1 and day 20 were diluted from antibody concentrations of 30 nM to 0.03 nM as calculated from the initial mixture for ELISA. Anti-human IgG (Fab specific)-peroxidase (Sigma-Aldrich) was used as the detection antibody.

### Cytotoxicity assay

The cytotoxicity of nCoVmab1 and nCoVmab2 to various cell lines, including L-929, HEp-2, OVCAR-3, Huh7, and Vero, was evaluated. Five types of cells were seeded in 96-well cell culture plates overnight. The IgGs (diluted with the growth medium) were added to the cells at a final concentration of 5 μM. TNFα, as a positive control, was added to L-929 cells, and PBS without protein was used as a negative control. After culturing at 37 °C for 24 h, 10 μl of Cell Counting Kit-8 (CCK-8) solution (Beyotime) was added following the manufacturer’s instructions. The absorbance was measured at 450 nm on a Synergy 2 Multi-Mode Reader (BioTek).

### Autoreactivity analysis of nCoVmab1 and nCoVmab2

The reactivity of nCoVmab1 and nCoVmab2 to HEp-2 cells was tested by indirect immunofluorescence. HEp-2 cells were seeded in 96-well culture plates overnight. The IgGs at concentrations of 25 μg/ml (diluted in PBS) was added to the cells, and 25 μg/ml 2F5 and PBS alone were used as the positive and negative controls, respectively. After incubating at 37 °C for 2 h and washing with PBS, FITC-mouse anti-human Fc (Cohesion Biosciences) was added and incubated for 1 h at 37 °C. After washing with PBS, fluorescence staining was observed by a fluorescence microscope (Leica).

### Animal protection experiments

hACE2 transgenic mice were purchased from GemPharmatech. Twenty-five male hACE2 transgenic mice (8–10 weeks old) were divided into five groups with five mice in each group to evaluate the efficacy of nCoVmab1 in prophylaxis and therapy. The mice were anesthetized with tribromoethanol (Avertin) and then intranasally inoculated with 1 × 10^4^ TCID_50_ SARS-CoV-2 in 30 μl of DMEM. Twelve hours before and 12 h after the viral challenge, the mice received an intraperitoneal dose of 5 or 20 mg/kg in a volume of 100 μl. An equivalent volume of PBS was administered as control after 12 h of the viral challenge.

All mice were euthanized at 3 dpi. The mouse lungs were homogenized with DMEM, clarified by centrifugation and supernatant was 10-fold diluted. A total of 200 μl supernatant diluent was added in monolayer Vero E6 cells in a 24-well plate for the determination of viral titers. Plaques were observed after 0.5% crystal violet staining.

### Histological analysis

The lung samples were fixed with 4% paraformaldehyde for 72 h, paraffin-embedded, and cut into 3.5-μm sections. Some tissue samples were stained with H&E for histopathological analysis whereas others were analyzed by indirect immunofluorescence assays (IFAs) for the detection of the SARS-CoV-2 antigen. For the IFAs, paraffin sections were firstly dewaxed and rehydrated as usual. Subsequently, the sections were soaked in the heated EDTA (pH 8.0) buffer for antigen retrieval. The slides were permeated with PBS/0.02% Triton X-100 for 15 min and then blocked with 5% BSA at room temperature for 1 h. The sections were incubated with a primary antibody (rabbit anti-2019-nCoV NP protein polyclonal antibody, 1:800, made in-house) for 1 h at 37 °C^21^. After washing in PBS, the tissues were incubated with Cy3-conjugated goat-antirabbit IgG (Abcam) at 1:200 dilution. After washing, the tissues were stained with DAPI (Beyotime) at 1:100 dilution. The tissue sections were cleared and mounted with neutral gum. The image collections were performed by Pannoramic MIDIsystem (3DHISTECH, Budapest) and FV1200 confocal microscopy (Olympus).

### Binding to chimeric RBDs

The epitopes of nCoVmab1 and nCoVmab2 were first refined by testing their binding to chimeric RBD-Fc fusions. The purified SARS-CoV-2 RBD-Fc and chimeric RBD-Fc fusions (RBD_SV_-RBM_SV-2_-Fc and RBD_SV-2_-RBM_SV_-Fc) were coated on a plate (Corning) at a concentration of 4 μg/ml overnight at 4 °C. Fivefold serially diluted antibodies were added to the wells. The following procedures were performed as described above.

### Competitive ELISA assay for binding site analysis

A competitive ELISA was used to further analyze the relationship of the binding sites of nCoVmab1 and nCoVmab2. The RBD was coated on the plate overnight at 4 °C. nCoVFab1 and nCoVFab2 at fixed final concentrations of 10 nM and 30 nM, respectively, were mixed with fivefold serially diluted competitive IgGs (nCoVFab1 vs. nCoVmab2 and nCoVFab2 vs. nCoVmab1, at concentrations from 200 to 0.0128 nM) and added to the RBD-coated wells. The bound Fabs were detected by an HRP-conjugated mouse anti-FLAG tag antibody (Sigma-Aldrich). The ELISA procedures were performed as described above.

### Binding to SARS-CoV-2 RBD mutants for mapping epitope by alanine scanning

RBD mutants including G446A, Y449A, Y453A, L455A, F456A, F486A, N487A, Y489A, Q493A, G496A, Q498A, T500A, N501A, G502A, Y505A were expressed and purified as Fc fusion proteins according to the methods described above. The binding of ACE2, nCoVmab1, and nCoVmab2 to these mutants was also performed by ELISA as described above.

### Germline analysis

Here, we analyzed the germline sequences of nCoVmab1 and nCoVmab2 with IMGT/V-QUEST. Then, we converted nCoVmab1 to its germline format with different heavy and light chain combinations as follows: nCoVmab1_gHgL_ (germline heavy chain and germline light chain), nCoVmab1_mHgL_ (mature heavy chain and germline light chain), and nCoVmab1_gHmL_ (germline heavy chain and mature light chain). In addition, we construed a nCoVmab1 mutant (nCoVmab1_gFR_) with the conversion of framework regions (FRs) to the corresponding germline sequence and reservation of the CDRs as their mature status. We also constructed serial germline formats for nCoVmab2 as we did for nCoVmab1. The binding ability of the purified germline antibodies to the RBD was tested and compared with that of the parental antibodies with an ELISA. The procedures were performed as described above.

### Statistical analysis

PRISM™ 8.0.2 for Windows (GraphPad) was used for the statistical analysis. To compare the experimental group with the control group, One-way ANOVA with Dunnett’s multiple comparisons was performed to determine significant differences. Statistical significance: ns, not significant, *P < 0.05, **P < 0.01. The median values were presented.

### Reporting summary

Further information on research design is available in the Nature Research Reporting Summary linked to this article.

## Supplementary information

Supplementary Information File

Reporting Summary

## Data Availability

Data underlying Figs. 1a, 2a–e, 3b, 5a–b, 6a–b, Supplementary Figs. 3, 5, 6, 7, and 8a are provided as Source Data file. The sequence information of heavy and light chain variable domains of nCoVmab1 and nCoVmab2 has been presented in the supplementary materials, which allows the use of the antibody sequences for non-commercial purposes. All other data are available from the corresponding author upon reasonable requests. Source data are provided with this paper.

## Source data

Source Data

## References

[1] Coronaviridae Study Group of the International Committee on Taxonomy of V. (2020). The species Severe acute respiratory syndrome-related coronavirus: classifying 2019-nCoV and naming it SARS-CoV-2. Nat. Microbiol..

[2] Zhu N (2020). A Novel Coronavirus from Patients with Pneumonia in China, 2019. N. Engl. J. Med..

[3] Zhou P (2020). A pneumonia outbreak associated with a new coronavirus of probable bat origin. Nature.

[4] Wrapp D (2020). Cryo-EM structure of the 2019-nCoV spike in the prefusion conformation. Science.

[5] Shang J (2020). Structural basis of receptor recognition by SARS-CoV-2. Nature.

[6] Walls AC (2020). Structure, Function, and Antigenicity of the SARS-CoV-2 Spike Glycoprotein. Cell.

[7] Du L (2009). The spike protein of SARS-CoV–a target for vaccine and therapeutic development. Nat. Rev. Microbiol..

[8] Du L (2017). MERS-CoV spike protein: a key target for antivirals. Expert Opin. Ther. Targets.

[9] Zhu Z (2013). Human monoclonal antibodies as candidate therapeutics against emerging viruses and HIV-1. Virol. Sin..

[10] Lan J (2020). Structure of the SARS-CoV-2 spike receptor-binding domain bound to the ACE2 receptor. Nature.

[11] Wang Q (2020). Structural and functional basis of SARS-CoV-2 entry by using human ACE2. Cell.

[12] Korber B (2020). Tracking changes in SARS-CoV-2 spike: evidence that D614G increases infectivity of the COVID-19 virus. Cell.

[13] Fernandez A (2020). Structural impact of mutation D614G in SARS-CoV-2 spike protein: enhanced infectivity and therapeutic opportunity. ACS Med. Chem. Lett..

[14] Mansbach, R. A. et al. The SARS-CoV-2 Spike variant D614G favors an open conformational state. *Sci. Adv.* **7**, eabf3671 (2021). 10.1126/sciadv.abf3671 PMC8051874 33863729

[15] Lee CY (2021). Human neutralising antibodies elicited by SARS-CoV-2 non-D614G variants offer cross-protection against the SARS-CoV-2 D614G variant. Clin. Transl. Immunol..

[16] Fenger M (2004). Detection of antinuclear antibodies by solid-phase immunoassays and immunofluorescence analysis. Clin. Chem..

[17] Zwick MB (2001). Broadly neutralizing antibodies targeted to the membrane-proximal external region of human immunodeficiency virus type 1 glycoprotein gp41. J. Virol..

[18] Haynes BF (2005). Cardiolipin polyspecific autoreactivity in two broadly neutralizing HIV-1 antibodies. Science.

[19] Winkler, E. S. et al. SARS-CoV-2 infection of human ACE2-transgenic mice causes severe lung inflammation and impaired function. *Nat. Immunol*. **21**, 1327–1335 (2020). 10.1038/s41590-020-0778-2 PMC7578095 32839612

[20] Jiang RD (2020). Pathogenesis of SARS-CoV-2 in transgenic mice expressing human angiotensin-converting enzyme 2. Cell.

[21] Shi R (2020). A human neutralizing antibody targets the receptor-binding site of SARS-CoV-2. Nature.

[22] Wu Y (2020). A noncompeting pair of human neutralizing antibodies block COVID-19 virus binding to its receptor ACE2. Science.

[23] Hurlburt NK (2020). Structural basis for potent neutralization of SARS-CoV-2 and role of antibody affinity maturation. Nat. Commun..

[24] Brochet X, Lefranc MP, Giudicelli V (2008). IMGT/V-QUEST: the highly customized and integrated system for IG and TR standardized V-J and V-D-J sequence analysis. Nucleic Acids Res..

[25] Giudicelli V, Brochet X, Lefranc MP (2011). IMGT/V-QUEST: IMGT standardized analysis of the immunoglobulin (IG) and T cell receptor (TR) nucleotide sequences. Cold Spring Harb. Protoc..

[26] Ge J (2021). Antibody neutralization of SARS-CoV-2 through ACE2 receptor mimicry. Nat. Commun..

[27] Bertoglio, F. et al. A SARA-CoV-2 neutralizing antibody selected from COVID-19 patients binds to the ACE2-RBD interface and is tolerant to most known RBD mutations. *Cell Rep* **36**, 109433 (2021). 10.1016/j.celrep.2021.109433 PMC8260561 34273271

[28] Rogers TF (2020). Isolation of potent SARS-CoV-2 neutralizing antibodies and protection from disease in a small animal model. Science.

[29] Wu NC (2020). An alternative binding mode of IGHV3-53 antibodies to the SARS-CoV-2 receptor binding domain. Cell Rep..

[30] Barnes CO (2020). Structures of human antibodies bound to SARS-CoV-2 spike reveal common epitopes and recurrent features of antibodies. Cell.

[31] Jiang, S., Zhang, X. & Du, L. Therapeutic antibodies and fusion inhibitors targeting the spike protein of SARS-CoV-2. *Expert Opin. Ther. Targets* 1–7 (2020). 10.1080/14728222.2020.1820482 PMC7544964 32941780

[32] Tuccori M (2020). Anti-SARS-CoV-2 neutralizing monoclonal antibodies: clinical pipeline. MAbs.

[33] Gottlieb RL (2021). Effect of Bamlanivimab as monotherapy or in combination with etesevimab on viral load in patients with mild to moderate COVID-19: a randomized clinical trial. JAMA.

[34] Weinreich DM (2021). REGN-COV2, a neutralizing antibody cocktail, in outpatients with Covid-19. N. Engl. J. Med..

[35] Xu J (2019). Antibodies and vaccines against Middle East respiratory syndrome coronavirus. Emerg. Microbes Infect..

[36] Coughlin MM, Prabhakar BS (2012). Neutralizing human monoclonal antibodies to severe acute respiratory syndrome coronavirus: target, mechanism of action, and therapeutic potential. Rev. Med. Virol..

[37] Ying T (2015). Junctional and allele-specific residues are critical for MERS-CoV neutralization by an exceptionally potent germline-like antibody. Nat. Commun..

[38] Zhang S (2018). Structural definition of a unique neutralization epitope on the receptor-binding domain of MERS-CoV spike glycoprotein. Cell Rep..

[39] Pan X (2020). Immunoglobulin fragment F(ab’)2 against RBD potently neutralizes SARS-CoV-2 in vitro. Antivir. Res..

[40] Dai L (2020). A universal design of betacoronavirus vaccines against COVID-19, MERS, and SARS. Cell.

[41] Richmond P (2021). Safety and immunogenicity of S-Trimer (SCB-2019), a protein subunit vaccine candidate for COVID-19 in healthy adults: a phase 1, randomised, double-blind, placebo-controlled trial. Lancet.

[42] Keech C (2020). Phase 1-2 trial of a SARS-CoV-2 recombinant spike protein nanoparticle vaccine. N. Engl. J. Med..

[43] Yang, S. et al. Safety and immunogenicity of a recombinant tandem-repeat dimeric RBD-based protein subunit vaccine (ZF2001) against COVID-19 in adults: two randomised, double-blind, placebo-controlled, phase 1 and 2 trials. *Lancet Infect. Dis* **21**, 1107–1119 (2021). 10.1016/S1473-3099(21)00127-4 PMC7990482 33773111

[44] Dallenbach K (2015). Protective effect of a germline, IL-17-neutralizing antibody in murine models of autoimmune inflammatory disease. Eur. J. Immunol..

[45] Schrade, A. et al. Back-to-Germline (B2G) procedure for antibody devolution. *Antibodies* **8**, 45 (2019). 10.3390/antib8030045 PMC6784197 31544851

[46] Zeng F (2018). Comprehensive elucidation of the structural and functional roles of engineered disulfide bonds in antibody Fc fragment. J. Biol. Chem..

[47] Zhu Z, Dimitrov DS (2009). Construction of a large naive human phage-displayed Fab library through one-step cloning. Methods Mol. Biol..

[48] Whitt MA (2010). Generation of VSV pseudotypes using recombinant DeltaG-VSV for studies on virus entry, identification of entry inhibitors, and immune responses to vaccines. J. Virol. Methods.

[49] Navarro D (1993). Glycoprotein B of human cytomegalovirus promotes virion penetration into cells, transmission of infection from cell to cell, and fusion of infected cells. Virology.

[50] Wang M (2020). Remdesivir and chloroquine effectively inhibit the recently emerged novel coronavirus (2019-nCoV) in vitro. Cell Res..

