# Supplementary material for: Identification of potent human neutralizing antibodies against SARS-CoV-2 implications for development of therapeutics and prophylactics
Source: Nat Commun. 2021 Aug 9;12:4887. doi: 10.1038/s41467-021-25153-x (PMC8352940; doi:10.1038/s41467-021-25153-x)
Supplement: Supplementary file 1 — Supplementary Information File [file 41467_2021_25153_MOESM1_ESM.pdf]

Supplementary Information

Identification of potent human neutralizing antibodies against SARS-CoV-2: implications for development of therapeutics and prophylactics

Zhao *et al*.

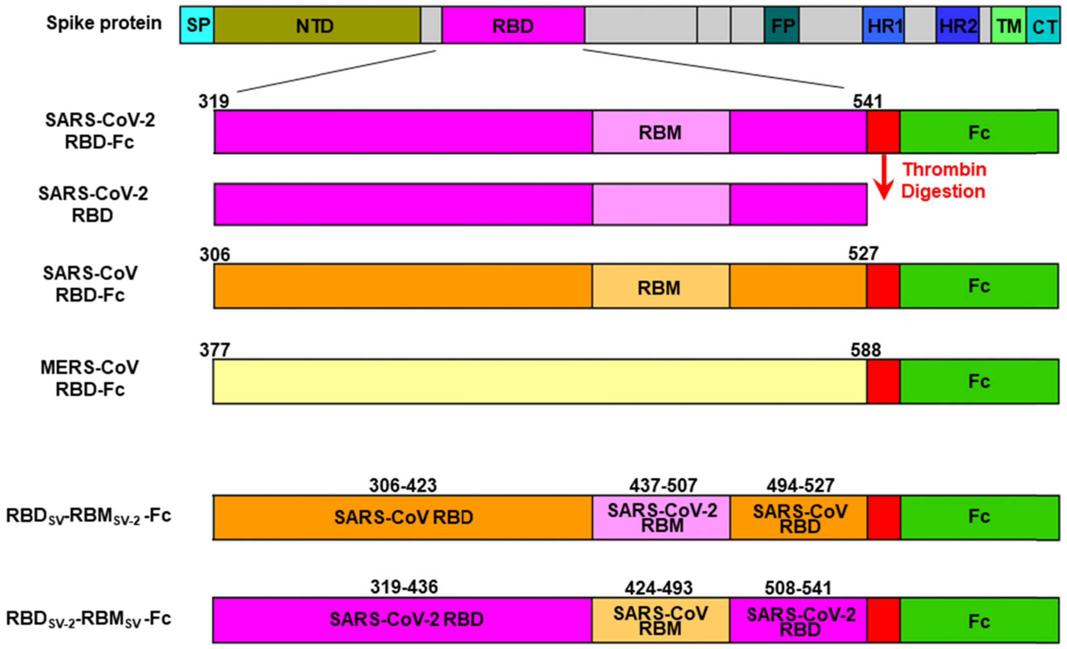

Supplementary Figure 1. Construction of RBDs from different coronaviruses (CoVs). The CoV envelope protein (spike protein) contains a signal peptide (SP), N-terminal domain (NTD), receptor-binding domain (RBD) with a receptor-binding motif (RBM), fusion peptide (FP), heptad repeat 1 (HR1), heptad repeat 2 (HR2), transmembrane region/domain (TM), and cytoplasmic tail (CT). The RBD was fused with an antibody Fc fragment for the construction of RBD-Fc. The SARS-CoV-2 RBD-Fc could be digested by thrombin to obtain an isolated RBD. The chimeric RBDs, including RBD_{SV}–$RBM_{SV\text{-}2}$-Fc and $RBD_{SV\text{-}2}$-RBM_{SV}-Fc, were also constructed with the replacement of RBMs.

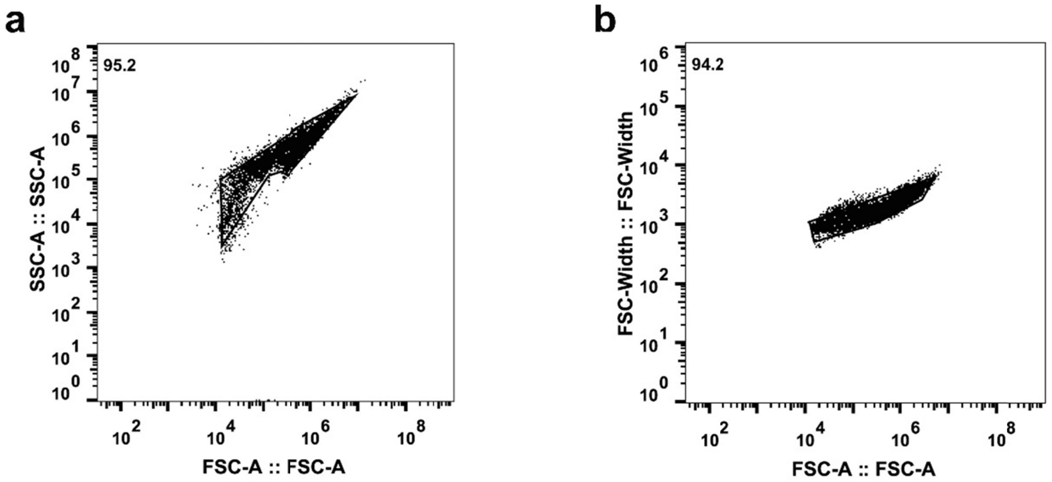

Supplementary Figure 2. Gating strategy for cell analysis. Cells were gated by flow cytometry **(a)** and cell aggregates (adherent cells) were excluded by FSC-A/FSC-Width **(b)**.

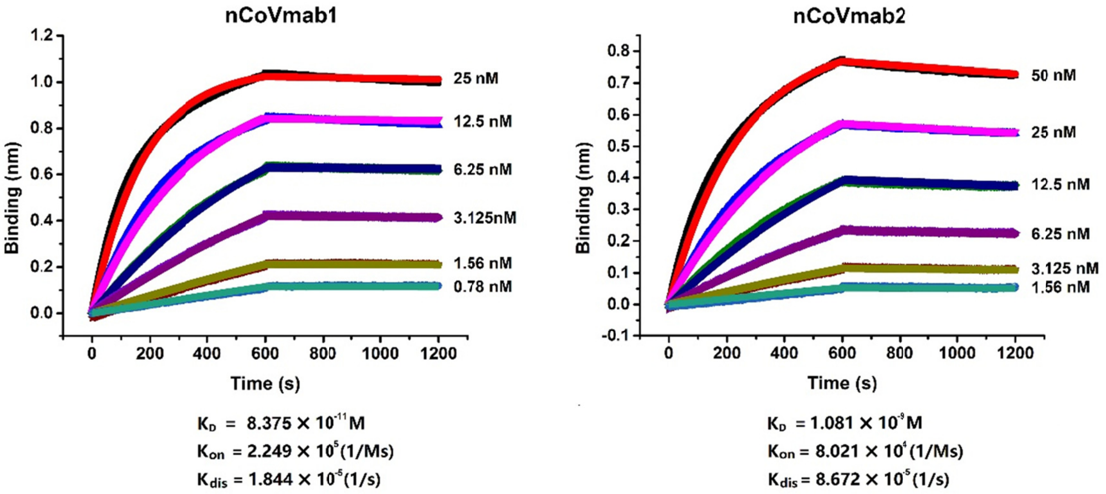

Supplementary Figure 3. Measurement of binding kinetics. The binding of SARS-CoV-2 RBD to biotinylated nCoVmab1 and nCoVmab2 was measured by BLI. Biotinylated nCoVmab1 and nCoVmab2 were loaded on the biosensors respectively. Serial diluted SARS-CoV-2 RBD was used for testing binding. Fast association and slow dissociation are observed. Source data are provided as a Source Data file.

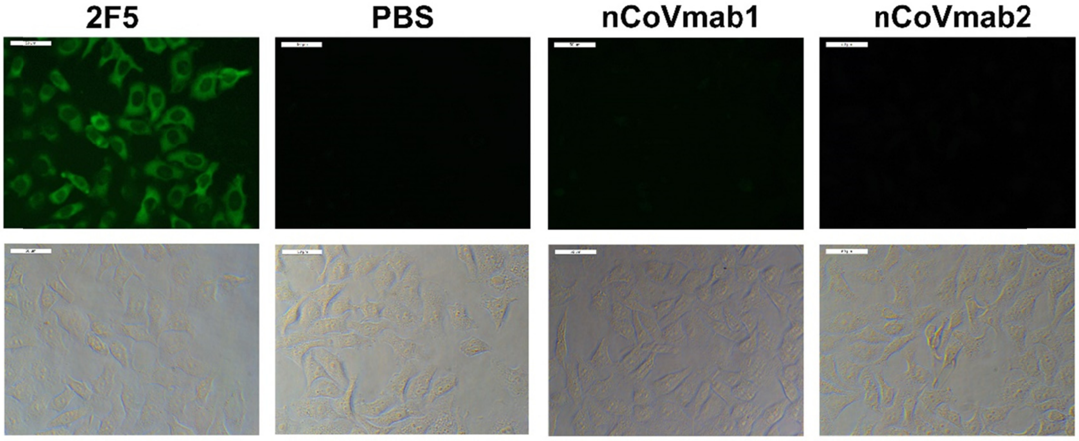

Supplementary Figure 4. Analysis of nCoVmab1 and nCoVmab2 autoreactivity. The binding of antibodies to HEp-2 epithelial cells was tested. 2F5 was added as a positive control, and a secondary antibody alone (PBS) was used as a negative control. The antibody concentration was 25 μg/ml. All pictures were acquired with a fluorescence microscope and magnified by 40× (scale bars, 50 μm). Data was performed with three independent experiments. Experiment was performed with duplicate samples. One representative is shown.

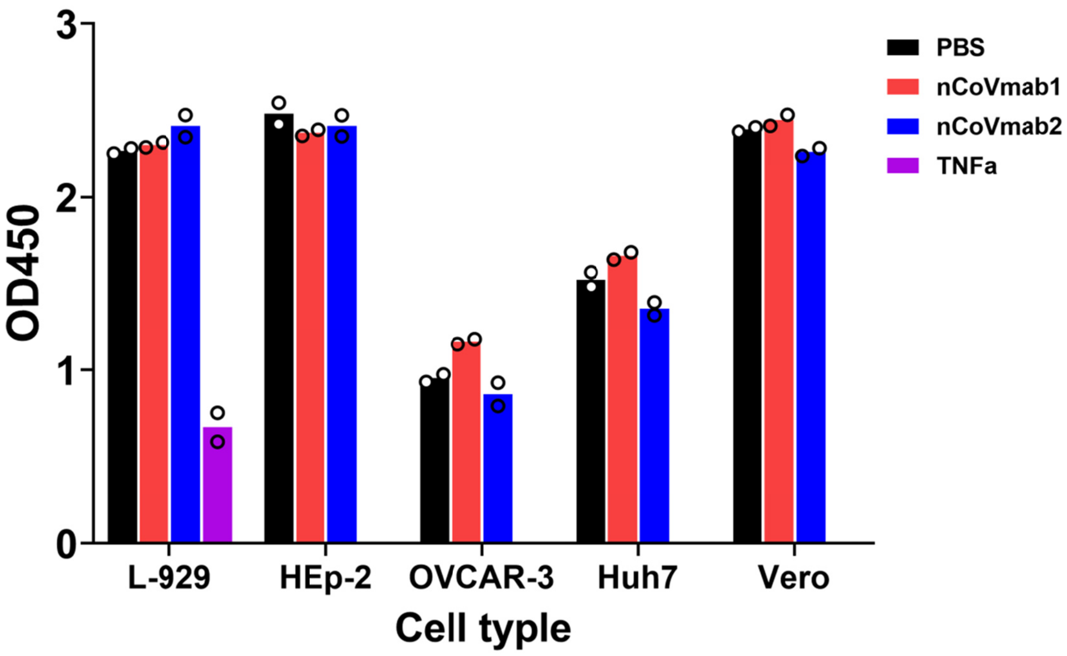

Supplementary Figure 5. Evaluation of the cytotoxicity of nCoVmab1 and nCoVmab2 to different cell lines. No detectable cytotoxicity was observed. By contrast, TNFα, as a positive control, was toxic to L-929 cells. Experiment was performed with duplicate samples. Source data are provided as a Source Data file.

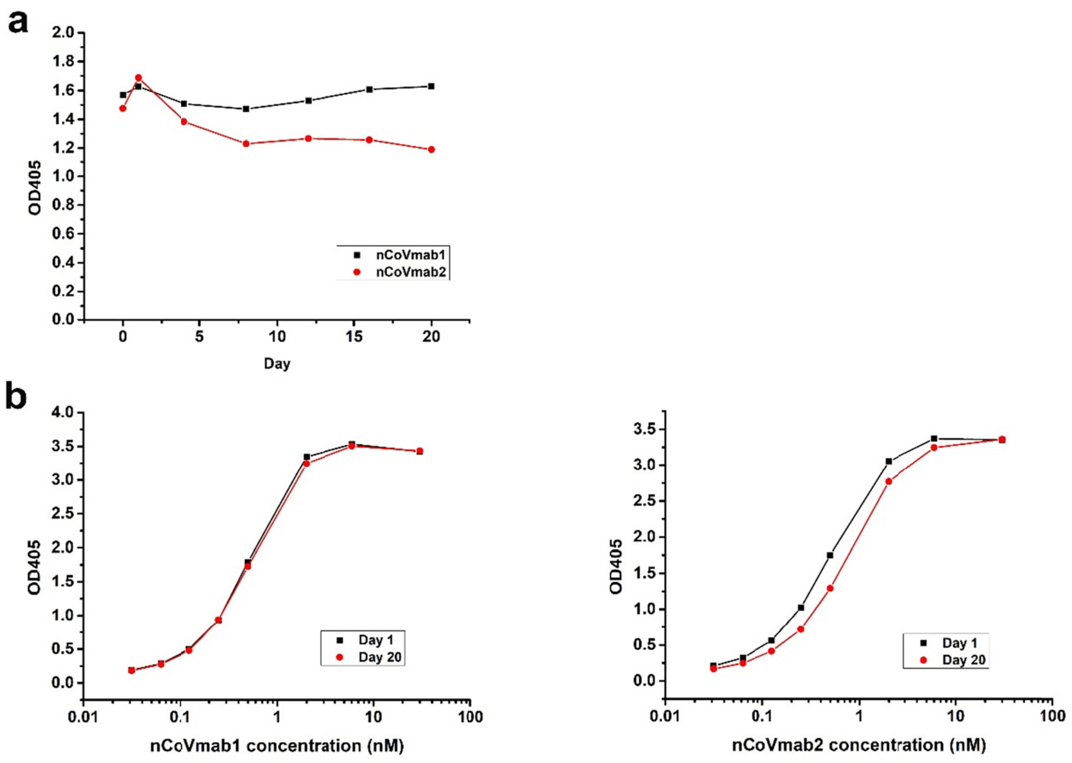

Supplementary Figure 6. Stability analysis of nCoVmab1 and nCoVmab2. (a) Samples from days 0, 1, 4, 8, 12, 16, and 20 were tested by binding to RBD-coated wells. The binding activity of nCoVmab2 slightly decreased compared with that of nCoVmab1. **(b)** Samples from days 1 and 20 were analyzed by an ELISA. No differences in the binding activities of nCoVmab1 were observed between the day 1 and day 20 samples, conversely, the binding ability of nCoVmab2 decreased after incubation. Experiment was performed with duplicate samples. Data are average values of two replicates. Source data are provided as a Source Data file.

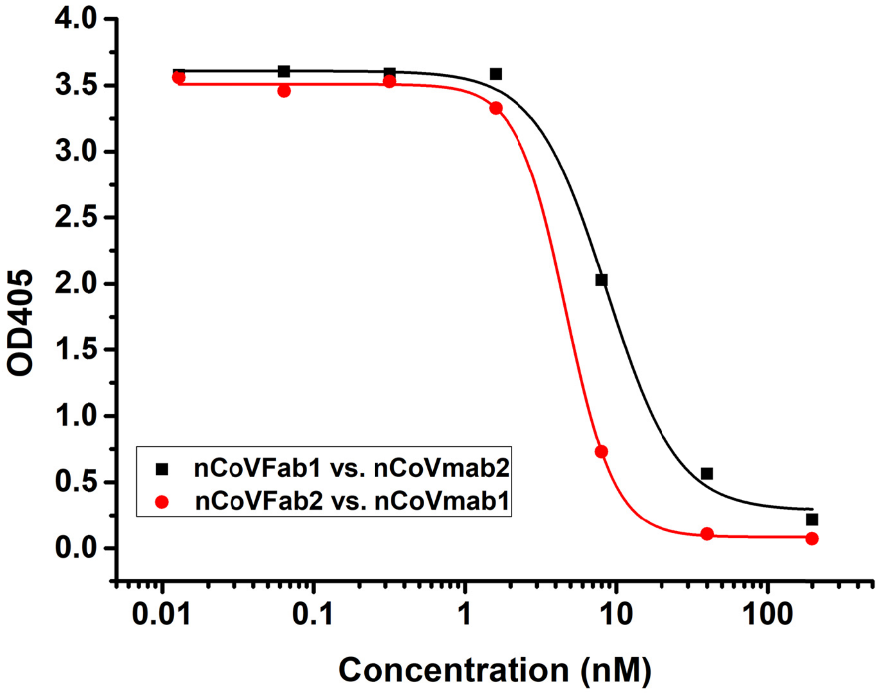

Supplementary Figure 7. Competitive binding analysis between nCoVmab1 and nCoVmab2. The competitive ELISA was used to analyze the relationship of the binding regions of the two antibodies. The black line represents competitive ELISA performed with fixed concentration of nCoVFab1 and diluted nCoVmab2. The red line represents competitive ELISA performed with a fixed concentration of nCoVFab2 and diluted nCoVmab1. They could almost completely compete with each other indicating that their epitopes highly overlap. Experiment was performed with duplicate samples. Data are average values of two replicates. Source data are provided as a Source Data file.

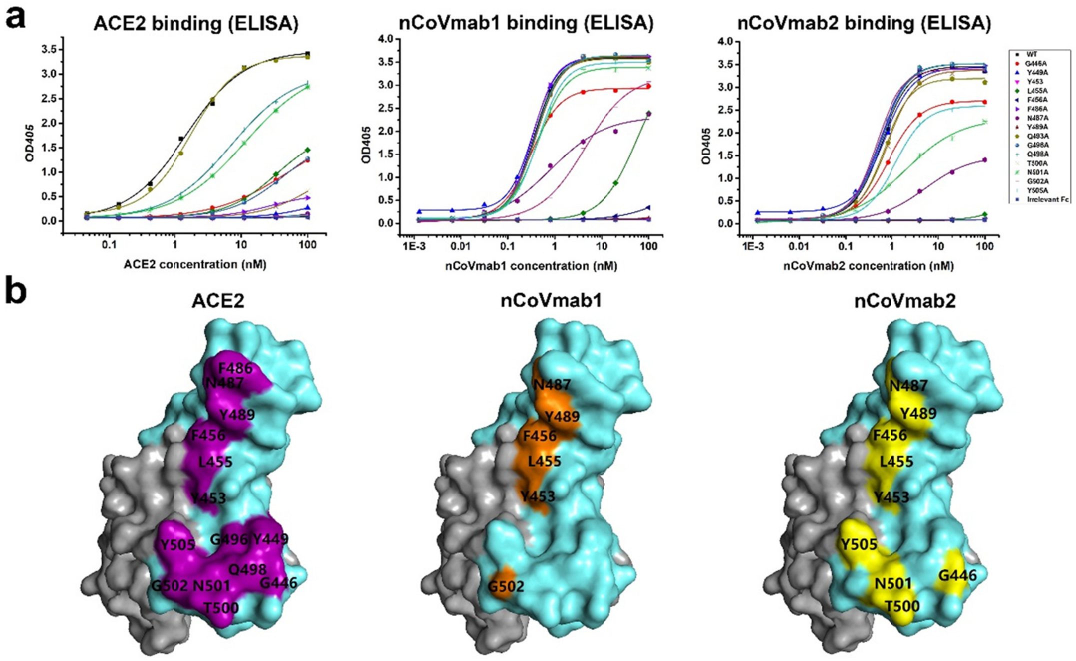

Supplementary Figure 8. Alanine scanning of key residues. (a) The binding of ACE2, nCoVmab1and nCoVmab2 to different SARS-CoV-2 RBD mutants was measured by ELISA. Experiment was performed with duplicate samples. Data are average values of two replicates. Source data are provided as a Source Data file. **(b)** Surface representation of residues in RBD involved in binding. The RBM region is denoted in cyan. The key residues for binding to hACE2, nCoVmab1 and nCoVmab2 are colored in purple, orange and yellow, respectively.

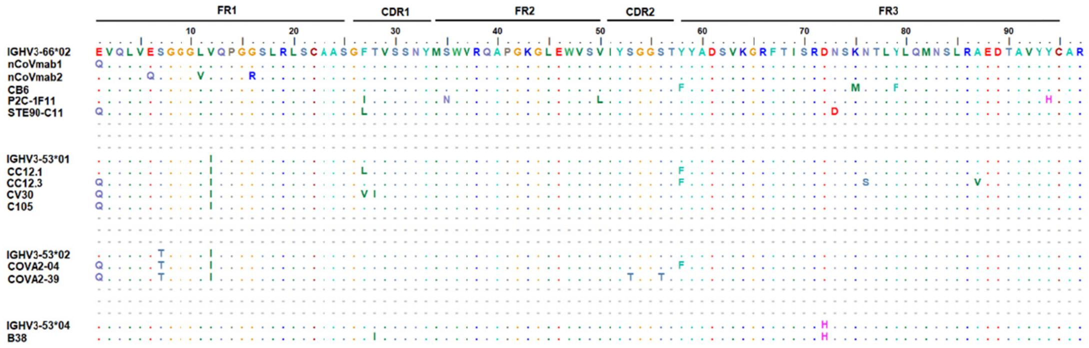

Supplementary Figure 9. Germline sequence alignment. Comparison of V_H sequences of nCoVmab1, nCoVmab2 and a panel reported antibodies with the corresponding germline sequences. CDR, complementarity-determining region; FR: framework.

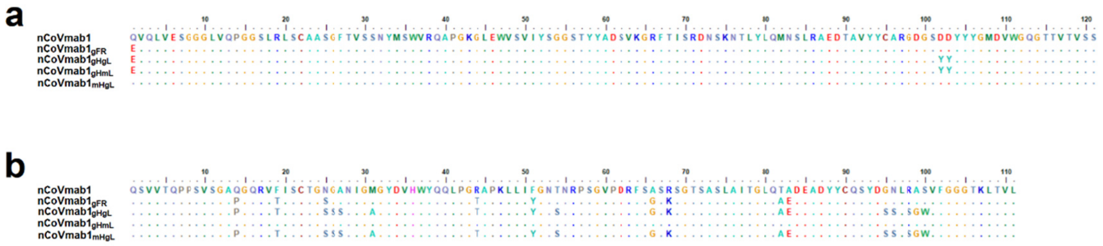

Supplementary Figure 10. Construction of different germline formats of nCoVmab1. (a) Heavy chain. **(b)** Light chain. nCoVmab1gFR, germline framework regions and mature CDRs; nCoVmab1$_{gHgL}$, germline heavy chain and germline light chain; nCoVmab1$_{gHmL}$, germline heavy chain and mature light chain; nCoVmab1$_{mHgL}$, mature heavy chain and germline light chain.

Supplementary Table 1. Microantiviral spread activity of nCoVmab1 and nCoVmab2 (I)

mAbs	Concentration (μg/ml)									Virus	Cell
	50	16.667	5.556	1.852	0.617	0.206	0.069	0.023	0.008		
nCoVmab1	-	-	-	-	+	++	+++	+++	++++	++++	-
	-	-	-	-	+	++	+++	++++	++++	++++	-
nCoVmab2	-	-	+	++	+++	++++	++++	++++	++++	++++	-
	-	-	+	++	+++	++++	++++	++++	++++	++++	-

Control	Concentration (μg/ml)							Virus	Cell
	4.821	2.410	1.205	0.603	0.301	0.151	0.075		
Remdesivir	-	-	+	++	++++	++++	++++	++++	-
	-	-	+	+++	++++	++++	++++	++++	-

"+" indicates that approximately 25% of cells in the well produced CPE caused by virus replication and spread, and "++++" indicates 100% CPE. "-" indicates that there was no virus plaque spread.

Supplementary Table 2. Microantiviral spread activity of nCoVmab1 and nCoVmab2 (II)

mAbs	Concentration (μg/ml)									Virus	Cell
	50	16.667	5.556	1.852	0.617	0.206	0.069	0.023	0.008		
nCoVmab1	-	-	-	-	-	**1**	+	++	++++	++++	-
	-	-	-	-	-	**2**	+	+++	++++	++++	-
nCoVmab2	-	-	-	**1**	+	++	+++	++++	++++	++++	-
	-	-	-	**5**	+	++	+++	++++	++++	++++	-

Control	Concentration (μg/ml)							Virus	Cell
	4.821	2.410	1.205	0.603	0.301	0.151	0.075		
Remdesivir	-	-	+	+++	+++	++++	++++	++++	-
	-	-	+	++	+++	++++	++++	++++	-

"+" indicates that approximately 25% of cells in the well produced CPE caused by virus replication and spread, and "++++" indicates 100% CPE. "-" indicates that there was no virus plaque spread.

Supplementary Table 3. Information of nCoVmab1, nCoVmab2 and a panel of reported antibodies

mAbs	V_H	Identity (V_H)	HCDR3	V_L	Identity (V_L)	LCDR3	IC_{50} (μg/ml)	Source
nCoVmab1	IGHV3-66*02	98.95%	ARGDGSDDYYYGMDV	IGLV1-40*02	90.97%	QSYDGNLRASV	0.010 (Live virus)	naïve phage library
nCoVmab2	IGHV3-66*02	98.25%	ARGDGSDDYYYGMDV	IGLV1-40*01	98.26%	QSYDSSLSGSV	0.139 (Live virus)	naïve phage library
CB6	IGHV3-66*01	99.0%	ARVLPMYGDYLDY	IGKV1-39*01	99.6%	QQSYSTPPEYT	0.036 (Live virus)	convalescent patient
P2C-1F11	IGHV3-66*02	95.9%	ARDLVVYGMDV	IGKV3-20*01	100.0%	QQYGSSPT	0.030 (pseudovirus)	convalescent patient
STE90-C11	IGHV3-66*02	98.6%	ARDVADAFDI	IGKV1-9*01	100%	QQLNSYPPFT	0.084 (Live virus)	convalescent patient
CC12.1	IGHV3-53*01	97.9%	ARDLDVYGLDV	IGKV1-9*01	97.9%	LNSYPPKFT	0.020 (pseudovirus)	convalescent patient
CC12.3	IGHV3-53*01	95.9%	ARDFGDFYFDY	IGKV3-20*01	99.0%	QQYGSSPRT	0.020 (pseudovirus)	convalescent patient
CV30	IGHV3-53*01	96.9%	ARDLDVSGGMDV	IGKV3-20*01	99.0%	QQYGSSPQT	0.030 (Live virus)	convalescent patient
C105	IGHV3-53*01	99.0%	ARGEGWELPYDY	IGLV2-8*01	97.0%	SSYEGSNNFVV	0.026 (pseudovirus)	convalescent patient
COVA2-04	IGHV3-53*02	97.9%	ARDLERAGGMDV	IGKV3-20*01	98.9%	QQYGSLYT	2.547 (Live virus)	convalescent patient
COVA2-39	IGHV3-53*02	96.9%	ARAHVDTAMVESGAFDI	IGLV2-23*02	98.0%	CSYAGSSTWV	0.054 (Live virus)	convalescent patient
B38	IGHV3-53*04	99.0%	AREAYGMDV	IGKV1-9*01	97.9%	QQLNSYPPYT	0.177 (Live virus)	convalescent patient
