# Supplementary material for: Identification of potent human neutralizing antibodies against SARS-CoV-2 implications for development of therapeutics and prophylactics
Source: Nat Commun. 2021 Aug 9;12:4887. doi: 10.1038/s41467-021-25153-x (PMC8352940; doi:10.1038/s41467-021-25153-x)
Supplement: Supplementary file 2 — Reporting Summary [file 41467_2021_25153_MOESM2_ESM.pdf]

nature portfolio

Corresponding author(s): GR

Last updated by author(s): Jul 8, 2021

Reporting Summary

Nature Portfolio wishes to improve the reproducibility of the work that we publish. This form provides structure for consistency and transparency in reporting. For further information on Nature Portfolio policies, see our Editorial Policies and the Editorial Policy Checklist.

Statistics

For all statistical analyses, confirm that the following items are present in the figure legend, table legend, main text, or Methods section.

n/a	Confirmed	
☐	☒	The exact sample size (n) for each experimental group/condition, given as a discrete number and unit of measurement
☐	☒	A statement on whether measurements were taken from distinct samples or whether the same sample was measured repeatedly
☐	☒	The statistical test(s) used AND whether they are one- or two-sided<br>*Only common tests should be described solely by name; describe more complex techniques in the Methods section.*
☒	☐	A description of all covariates tested
☐	☒	A description of any assumptions or corrections, such as tests of normality and adjustment for multiple comparisons
☐	☒	A full description of the statistical parameters including central tendency (e.g. means) or other basic estimates (e.g. regression coefficient) AND variation (e.g. standard deviation) or associated estimates of uncertainty (e.g. confidence intervals)
☐	☒	For null hypothesis testing, the test statistic (e.g. F, t, r) with confidence intervals, effect sizes, degrees of freedom and P value noted<br>*Give P values as exact values whenever suitable.*
☒	☐	For Bayesian analysis, information on the choice of priors and Markov chain Monte Carlo settings
☒	☐	For hierarchical and complex designs, identification of the appropriate level for tests and full reporting of outcomes
☒	☐	Estimates of effect sizes (e.g. Cohen's d, Pearson's r), indicating how they were calculated

Our web collection on statistics for biologists contains articles on many of the points above.

Software and code

Policy information about availability of computer code

Data collection	CytoFLEX (Beckman Coulter, RRID:SCR_019627), Octet RED96 (ForteBio), Operetta CLS (PerkinElmer, RRID:SCR_018810), Gen5 CHS 2.04 (RRID:SCR_017317)
Data analysis	Data Alaysis 7.0 software<br>Harmony3.5 (PerkinElmer, RRID:SCR_018809)<br>Pannoramic MIDIsystem (3DHISTECH, Budapest, RRID:SCR_014424)<br>FV1200 confocal microscopy (Olympus, RRID:SCR_017564)<br>GraphPad PRISM™ 8.0.2 (RRID:SCR_002798)<br>FlowJo 7.6.5 (RRID:SCR_008520)

For manuscripts utilizing custom algorithms or software that are central to the research but not yet described in published literature, software must be made available to editors and reviewers. We strongly encourage code deposition in a community repository (e.g. GitHub). See the Nature Portfolio guidelines for submitting code & software for further information.

Data

Policy information about availability of data

All manuscripts must include a data availability statement. This statement should provide the following information, where applicable:

- Accession codes, unique identifiers, or web links for publicly available datasets
- A description of any restrictions on data availability
- For clinical datasets or third party data, please ensure that the statement adheres to our policy

Data underlying Figs. 1a, 2a-e, 3b, 5a-b, 6a-b, and Supplementary Figs. 3, 5, 6, 7 and 8a are provided as Source Data files. The sequence information of heavy and

light chain variable domains of nCoVmab1 and nCoVmab2 has been presented in the Supplementary material. which allows the use of the antibody sequences for non-commercial purposes. All other data are available from the corresponding author upon reasonable requests. Source data are provided with this paper.

Field-specific reporting

Please select the one below that is the best fit for your research. If you are not sure, read the appropriate sections before making your selection.

- [x] Life sciences
- [ ] Behavioural & social sciences
- [ ] Ecological, evolutionary & environmental sciences

For a reference copy of the document with all sections, see nature.com/documents/nr-reporting-summary-flat.pdf

Life sciences study design

All studies must disclose on these points even when the disclosure is negative.

Sample size	For FACS-based experiments, a standard number of 100000 cells/sample was gated by FSC/SSC and cell aggregates were excluded by FSC-A/ FSC-Width.<br>For viral infection experiments, infection dose of authentic virus infection experiments was based on Pfu to prevent double infections of cells, or on fluorescent plaques (for VSV pseudotyping exp.) to be in linear range of measurement.<br>For animal study, sample sizes were determined based on our pre-experiment in vivo. Use of 5 mice per group represents a minimally sufficient same sizes to produce more reliable results.
Data exclusions	No data were excluded from the analysis
Replication	Key experimental findings that include identification of neutralizing human monoclonal antibodies and antibody binding were confirmed in two or more independent experiments. Antibody blocking assay was confirmed by FACS.<br>Initial virus neutralization assay was confirmed using a VSV pseudotyped SARS-CoV-2, and the neutralization activity of these mAbs was confirmed using authentic virus SARS-CoV-2 in a BSL-3.<br>Antibodies in the competition experiment showed blocking effect.
Randomization	In the process of monoclonal identification, different colonies were randomly selected into different 96 well plates for protein expression and ELISA assays.
Blinding	The initial antibody expression and screening for antigen reactivity was done in a blinded fashion, as a given antibody sequence was not known to the investigator at time of analysis. Importantly, multiple antibodies discovered by independent workflows were closely related and some had identical amino acid sequences, and exhibited similar phenotypes in both antigen binding assays. For the antibody validation experiments, investigators were not blinded to the study groups.

Reporting for specific materials, systems and methods

We require information from authors about some types of materials, experimental systems and methods used in many studies. Here, indicate whether each material, system or method listed is relevant to your study. If you are not sure if a list item applies to your research, read the appropriate section before selecting a response.

Materials & experimental systems

n/a	Involved in the study	
[ ]	[x]	Antibodies
[ ]	[x]	Eukaryotic cell lines
[x]	[ ]	Palaeontology and archaeology
[ ]	[x]	Animals and other organisms
[x]	[ ]	Human research participants
[x]	[ ]	Clinical data
[x]	[ ]	Dual use research of concern

Methods

n/a	Involved in the study	
[x]	[ ]	ChIP-seq
[ ]	[x]	Flow cytometry
[x]	[ ]	MRI-based neuroimaging

Antibodies

Antibodies used	1. HRP-conjugated anti-M13 mouse monoclonal antibody (HRP) (Sino Biological) Catalog Number: 11973-MM05T-H<br>2. A mouse monoclonal ANTI-FLAG® M2-Peroxidase (HRP) antibody (Sigma-Aldrich) Catalog Number: A8592<br>3. Goat anti-human IgG Fc-DyLight 650 (Invitrogen) Catalog Number: SA5-10137<br>4. Anti-human IgG (Fab specific)-peroxidase (Sigma-Aldrich) Catalog Number: A0293<br>5. HRP-conjugated streptavidin (Proteintech) Catalog Number: SA00001-0<br>6. Cy3-conjugated goat-antirabbit IgG (Abcam) Catalog Number: ab97075<br>7. FITC-mouse anti-human Fc (Cohesion Biosciences) Catalog Number: CAS2338
Validation	All the secondary antibodies were bought from commercial vendors and were validated by the manufacturers.

Eukaryotic cell lines

Policy information about cell lines

Cell line source(s)	1. African green monkey origin, Vero, from ATCC<br>2. African green monkey origin, Vero E6, from CCTCC<br>3. Human embryonic kidney cell, 293T cells, from CCTCC<br>4. Mouse fibroblast, L-929, from CCTCC<br>5. Human laryngeal epidermoid carcinoma, HEp-2, from CCTCC<br>6. Human liver cancer cell, Huh7 cells, from CCTCC<br>7. Human ovarian adenocarcinoma, OVCAR-3 cells, from CCTCC<br>8. Human embryonic kidney cell, HEK293F cells, from Thermo
Authentication	All cell lines were previously reported but not authenticated by us.
Mycoplasma contamination	We confirm that all cells were tested as mycoplasma negative.
Commonly misidentified lines<br>(See ICLAC register)	No commonly misidentified cell lines were used.

Animals and other organisms

Policy information about studies involving animals; ARRIVE guidelines recommended for reporting animal research

Laboratory animals	For viral challenge, the authentic SARS-CoV-2, hACE2-KI/NIFDC mice (8-10 weeks, C57BL/6, male) were purchased from GemPharmatech. Animals were housed in groups of up to 5 mice/case at 18-24°C (ambient temperature) and 40-60% humidity. Mice were on a 20% protein diet and maintained on a 12-hour light/dark cycle (8 am and 8 pm). Food and water were available ad libitum.
Wild animals	No wild animals were used.
Field-collected samples	No field-collected samples were used.
Ethics oversight	All processes of the animal experiment were in line with recommendations for the care and use of laboratory animals and the Institutional Review Board of the Wuhan Institute of Virology, Chinese Academy of Sciences (Ethics number: WIVA21202002).

Note that full information on the approval of the study protocol must also be provided in the manuscript.

Flow Cytometry

Plots

Confirm that:

- [x] The axis labels state the marker and fluorochrome used (e.g. CD4-FITC).
- [x] The axis scales are clearly visible. Include numbers along axes only for bottom left plot of group (a 'group' is an analysis of identical markers).
- [x] All plots are contour plots with outliers or pseudocolor plots.
- [x] A numerical value for number of cells or percentage (with statistics) is provided.

Methodology

Sample preparation	Vero E6 cells were disrupted with sodium citrate (dissolved in PBS) and aliquoted into tubes. The Fabs (final concentration: 100 μg/ml) were incubated with RBD-Fc(final concentration: 2.5 μg/ml) at 4°C for 1 h, and then the mixture was added to 60 μl of cells for incubation at 4°C for 1.5 h. The cells were washed once with PBS. Goat anti-human IgG Fc-DyLight 650 (Invitrogen) was used as a secondary antibody for incubation at 4°C for 1 h. The cells were washed again with PBS. Finally, the cells were resuspended in 200 μl of PBS for analysis.
Instrument	Flow cytometry was performed using CytoFLEX LX Flow Cytometer, Beckman Coulter
Software	Data were analyzed by FlowJo 7.6.5.
Cell population abundance	All cells are just one type of cell. Cell population abundance is not applicable in the study.
Gating strategy	Cells were gated by FSC/SSC. Cells aggregates (adherent cells) were excluded by FSC-A/FSC-Width.

- [x] Tick this box to confirm that a figure exemplifying the gating strategy is provided in the Supplementary Information.
